# Endogenous Annexin-A1 Regulates Haematopoietic Stem Cell Mobilisation and Inflammatory Response Post Myocardial Infarction in Mice *In Vivo*

**DOI:** 10.1038/s41598-017-16317-1

**Published:** 2017-11-30

**Authors:** Cheng Xue Qin, Siobhan B. Finlayson, Annas Al-Sharea, Mitchel Tate, Miles J. De Blasio, Minh Deo, Sarah Rosli, Darnel Prakoso, Colleen J. Thomas, Helen Kiriazis, Eleanor Gould, Yuan H. Yang, Eric F. Morand, Mauro Perretti, Andrew J. Murphy, Xiao-Jun Du, Xiao-Ming Gao, Rebecca H. Ritchie

**Affiliations:** 1 Baker Heart & Diabetes Institute, Melbourne, 3004 Australia; 2 Dept of Pharmacology and Therapeutics, University of Melbourne, Parkville, 3010 Australia; 3 Dept of Physiology, Anatomy and Microbiology, La Trobe University, Bundoora, 3086 Australia; 4 Dept of Medicine, Central Clinical School, Monash University, Melbourne, 3004 Australia; 5 School of Biosciences, University of Melbourne, Parkville, 3010 Australia; 6 Centre for Inflammatory Diseases, Monash University, Clayton, 3168 VIC, Australia; 7 Department of Immunology, Monash University, Melbourne, 3004 VIC, Australia; 8 William Harvey Research Institute, Barts and The London School of Medicine, Queen Mary University of London, London, United Kingdom

## Abstract

Endogenous anti-inflammatory annexin-A1 (ANX-A1) plays an important role in preserving left ventricular (LV) viability and function after ischaemic insults *in vitro*, but its long-term cardioprotective actions *in vivo* are largely unknown. We tested the hypothesis that ANX**-**A1-deficiency exaggerates inflammation, haematopoietic stem progenitor cell (HSPC) activity and LV remodelling in response to myocardial ischaemia *in vivo*. Adult *ANX****-****A1*^−/−^ mice subjected to coronary artery occlusion exhibited increased infarct size and LV macrophage content after 24–48 h reperfusion compared with wildtype (WT) counterparts. In addition, *ANX****-****A1*^−/−^ mice exhibited greater expansion of HSPCs and altered pattern of HSPC mobilisation 8 days post-myocardial infarction, with increased circulating neutrophils and platelets, consistent with increased cardiac inflammation as a result of increased myeloid invading injured myocardium in response to MI. Furthermore, *ANX****-****A1*^−/−^ mice exhibited significantly increased expression of LV pro-inflammatory and pro-fibrotic genes and collagen deposition after MI compared to WT counterparts. ANX-A1-deficiency increased cardiac necrosis, inflammation, hypertrophy and fibrosis following MI, accompanied by exaggerated HSPC activity and impaired macrophage phenotype. These findings suggest that endogenous ANX**-**A1 regulates mobilisation and differentiation of HSPCs. Limiting excessive monocyte/neutrophil production may limit LV damage *in vivo*. Our findings support further development of novel ANX-A1-based therapies to improve cardiac outcomes after MI.

## Introduction

Current revascularisation approaches (thrombolytic therapy, percutaneous interventions) remain the most effective strategies for reducing cardiac damage after acute myocardial infarction (MI), yet MI and subsequent heart failure (HF) remain the leading cause of death worldwide^1^. Paradoxically, restoration of blood flow post-MI can induce further injury, termed ‘reperfusion injury’^2^. Local inflammation has been implicated as a key contributing mediator to the cardiac injury, retriggering subsequent adverse remodelling and progression to heart failure^3^. Pain and anxiety during MI stimulates mobilisation of haematopoietic stem and progenitor cells (HSPCs)^4^, which can promote unstable characteristics in previously-stable atherosclerotic lesions. This mobilisation of HSPCs is critical for the inflammatory response in MI. Therefore, strategies targeting HSPC activity may be therapeutically relevant in settings of MI.

Annexin-A1 (ANX-A1) is a glucocorticoid-regulated protein^5^ that has anti-inflammatory and pro-resolving functions^6,7^. This 37 kDa protein is ubiquitously expressed in various cell types in the body, including liver, lung, spleen and heart, with highest levels detected in neutrophils and macrophages^6^. Several lines of evidence indicate ANX-A1 also protects the myocardium from ischaemic injury, reducing infarct size^8–10^. This has been largely attributed to its anti-inflammatory actions^10,11^. ANX-A1 deficiency increases neutrophil migration and expression of pro-inflammatory cytokines, exacerbating inflammatory responses in several experimental models, including arthritis^12–14^, ulcerative colitis^15^, systemic lupus erythematosus^16^ and atherosclerosis^17^. In isolated mouse hearts, deficiency of ANX-A1 further impairs recovery of left ventricular (LV) function, and markedly reduces activities of the pro-cell survival kinase, Akt, following ischaemia-reperfusion (I-R) *in vitro*^8^. The cardioprotective and anti-inflammatory actions of endogenous ANX-A1 *in vivo*, beyond the first few hours after an ischaemic insult (and of greater relevance to the clinical context), are however yet to be elucidated. Further, the mechanisms by which ANX-A1 exerts its anti-inflammatory effects *in vivo* remain elusive. It is postulated that ANX-A1 is released from inflammatory cells, and binds to formyl peptide receptor-2 (FPR2) at the site of injury, preventing leukocyte adhesion and infiltration^7,18^. Given HSPCs are the precursors of innate inflammatory cells, and play an important role in the inflammatory response in MI, the effect of ANX-A1 on HSPC activity after an ischaemic insult clearly warrants further investigation^19^. Therefore, we explored the hypothesis that *ANX****-****A1*^−/−^ deficiency exaggerates both HSPC activity and the subsequent inflammatory response following myocardial ischaemia *in vivo*.

## Results

### ANX-A1 expression increases after I-R or MI

**Figure 1:**
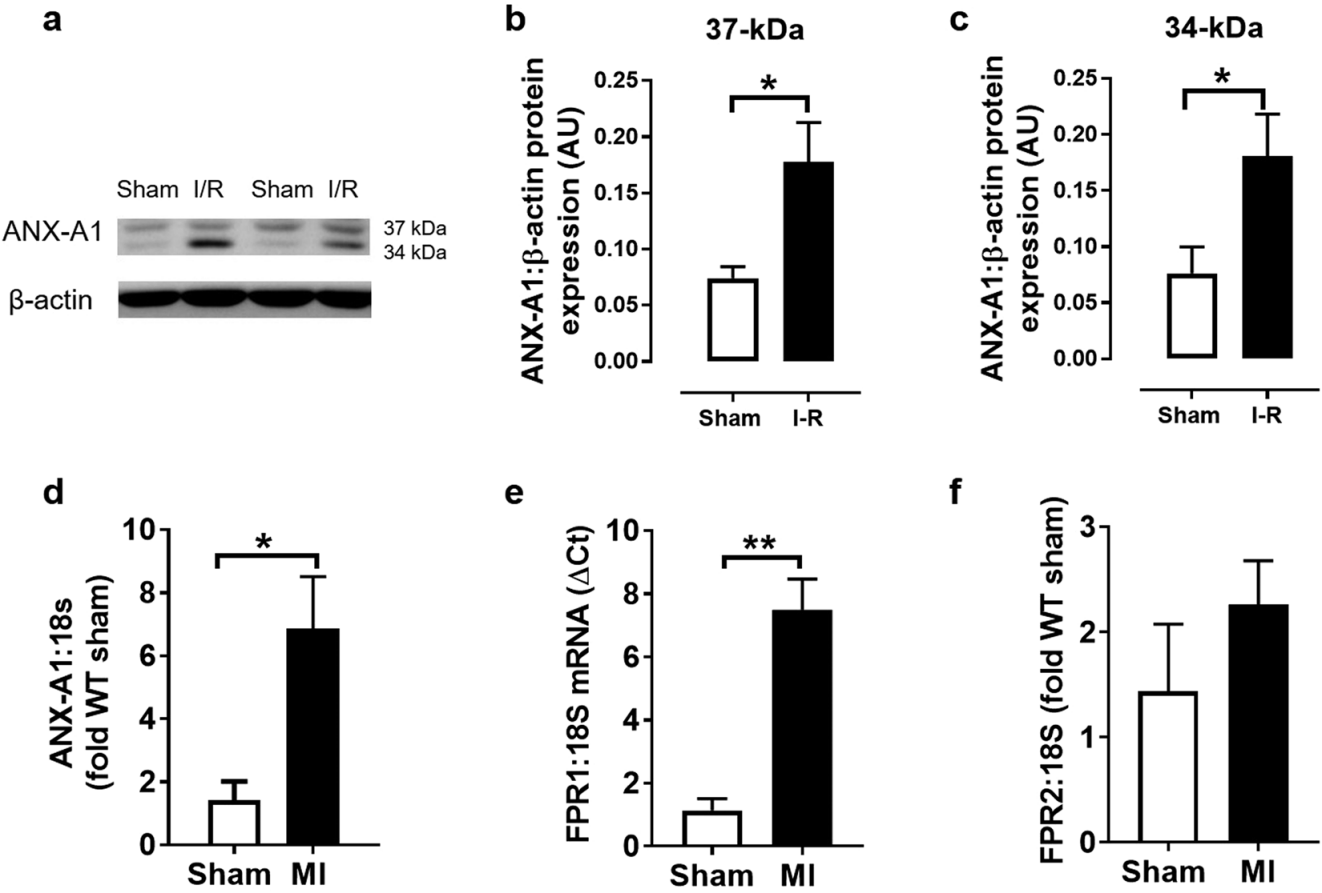
Expression of endogenous ANX-A1 increases after myocardial I-R or MI. (**a**) Representative western blot of endogenous ANX-A1 protein expression in isolated rat hearts subjected to sham or I-R *in vitro*. Densitometry analysis of the ANX-A1 isoform (**b**) 37 kDa and (**c**) 34 kDa isoforms (sham shown as white bars, or I-R as black bars). LV gene expression of (**d**) ANX-A1, (**e**) FPR1 and (**f**) FPR2 in mice subjected to myocardial infarction or sham operation *in vivo* (sham shown as white bars, or MI as black bars). The full-length blots are presented in Supplementary Figure 1a. Data is presented as mean ± SEM. *P < 0.05, **P < 0.01, vs. respective shams (unpaired student’s *t*-test, n = 5–6 per group).

To assess the influence of an ischaemic insult on the expression of ANX-A1 and its receptors (FPR1 and FPR2), firstly we examined protein levels of ANX-A1 in rodent hearts subjected to ischaemia and reperfusion in a Langendorff model. As expected, a small amount of ANX-A1 was detected in extracts from normoxic sham rat hearts *in vitro* (Fig. 1a–c). However, there was a significant increase in the protein levels of the ANX-A1 doublet (34 kDa and 37kD isoforms) in rat isolated hearts subjected to I-R *in vitro*. Permanent LAD occlusion in mice *in vivo* also induced significant increases in the gene expression of both ANX-A1 and FPR1 by almost 4 fold (Fig. 1d,e), but not in FPR2 (Fig. 1f).

### ANX-A1 deficiency exaggerates cardiac infarct size after I-R *in vivo*

**Figure 2:**
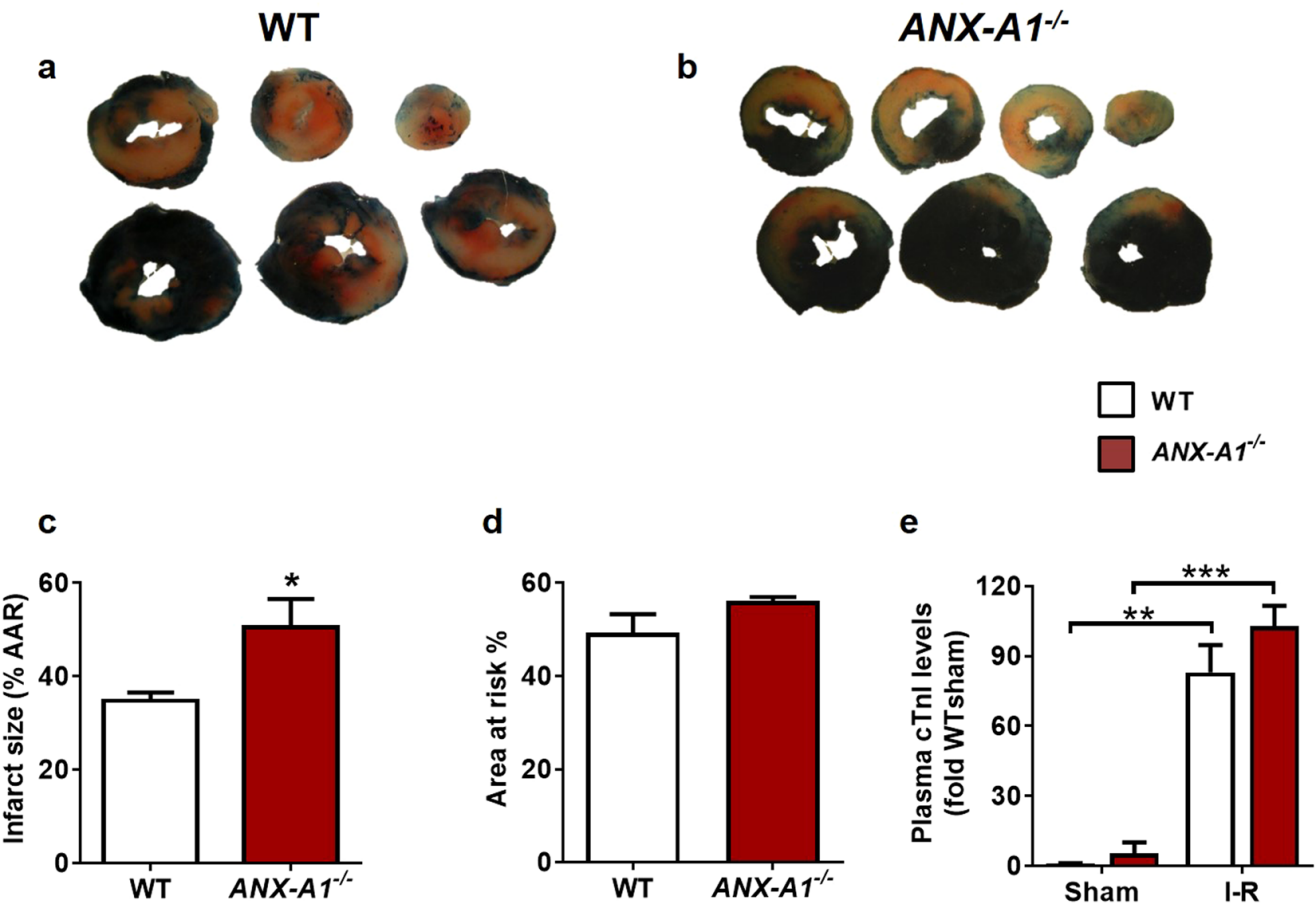
Deficiency of *ANX-A1* exacerbates myocardial injury after 1 h ischaemia and 24 h reperfusion. Representative LV sections from (**a**) WT and (**b**) *ANX-A1*^−/−^ mice 24 h after I-R injury. (**c**) Infarct size and (**d**) ischaemic zone (% area-at-risk, AAR) in WT (white bars) and *ANX-A1*^−/−^ mice (red bars). (**e**) Plasma cardiac troponin I (cTnI) levels. *P < 0.05, **P < 0.01, ***P < 0.001 vs. respective shams (unpaired Student’s *t*-test was used to analyse infarct size, n = 8–9 per group).

Adult male WT or *ANX-A1*^−/−^ mice were randomly assigned to either myocardial I-R injury, MI or sham procedures *in vivo*. Mice in cohorts 1 and 2 were then subjected to reperfusion for 24 h and 48 h respectively, which are optimal time points for the assessment of cardiac necrosis and early cardiac inflammation *in vivo*^20^. Animals in cohorts 3 and 4 were subjected to a more severe ischaemic insult, permanent LAD ligation for either 8 days or 4 weeks, respectively^21^, to assess the mobilisation of HSPCs, severity of regional inflammation and early cardiac remodelling (cohort 3), and on cardiac physiology over the longer-term (cohort 4). Sham animals underwent identical surgical procedures but without ligation. Following 1 h ischaemia/24 h reperfusion, infarct size (IS) was exaggerated in *ANX-A1*^−/−^ compared with WT mice (P < 0.05, Fig. 2a–c), despite similar area at risk (AAR, Fig. 2c). Myocardial injury was also quantitatively assessed at 24 h reperfusion using plasma levels of cardiac troponin I (cTnI), which were clearly elevated in both MI groups compared with sham, but no difference was observed between genotypes (Fig. 2e).

### ANX-A1 deficiency exaggerates inflammatory cells infiltration after I-R *in vivo*

**Figure 3:**
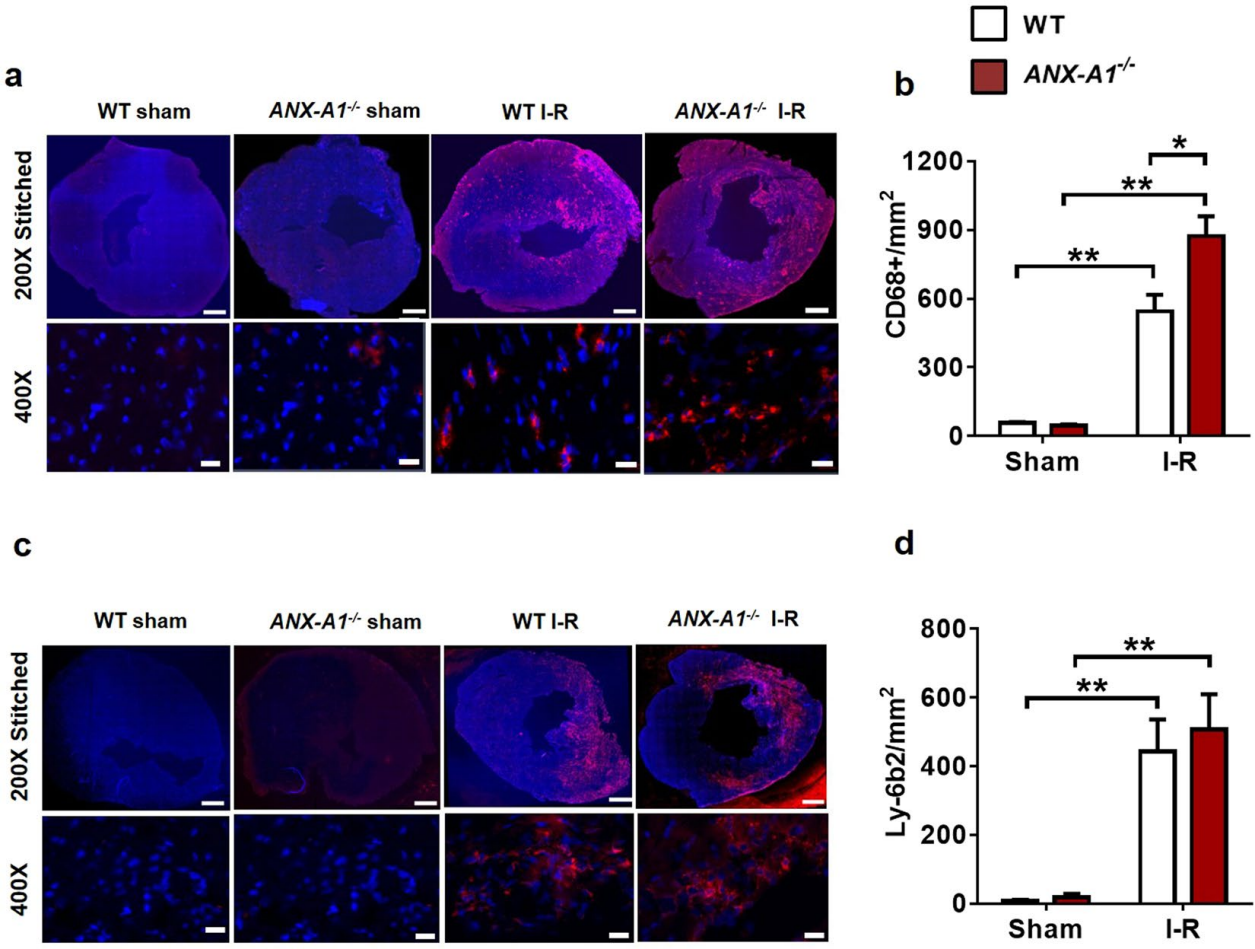
Deficiency of ANX-A1 exacerbates inflammatory cell infiltration after myocardial injury. (**a**,**b**) Cardiac macrophage and (**c**,**d**) neutrophil detection. Representative LV images 48 h after I-R injury acquired by fluorescence microscopy with (**a**) anti-CD68^+^ and (**c**) anti-Ly6b.2. Higher magnification images reveals overlay of dark blue (DAPI; detecting nuclei) and red (macrophages or neutrophils, respectively) indicating positive staining (scale-bar in top panels = 500 µm; in bottom panels = 100 µm). Quantification of (**b**) macrophages and (**d**) neutrophils. Data is presented as mean ± SEM, *P < 0.05, **P < 0.01, vs. respective shams (two-way ANOVA followed by Tukey’s post-hoc test). Shams n = 3, I-R n = 5–6/group, with WT and *ANX-A1*^−/−^ mice shown in white and red bars, respectively.

The impact of ANX-A1 deficiency on inflammatory cell infiltration into the ischaemic myocardium was examined 48 h post I-R (Fig. 3a–d). Macrophage density (CD68-positive cells) was markedly increased following 48 h reperfusion in both genotypes (P < 0.01 vs. sham; Fig. 3a,c). This was even more pronounced in *ANX-A1*^−/−^ mice (by > 40%), compared with WT after I-R (P < 0.05, Fig. 3a,c). Neutrophil density (Ly-6b2-positive cells) was also considerably increased after 48 h reperfusion (P < 0.01 vs. sham), but to a similar level in both genotypes (Fig. 3b,d).

### ANX-A1 deficiency exaggerates HSPC mobilisation post-MI *in vivo*

**Figure 4:**
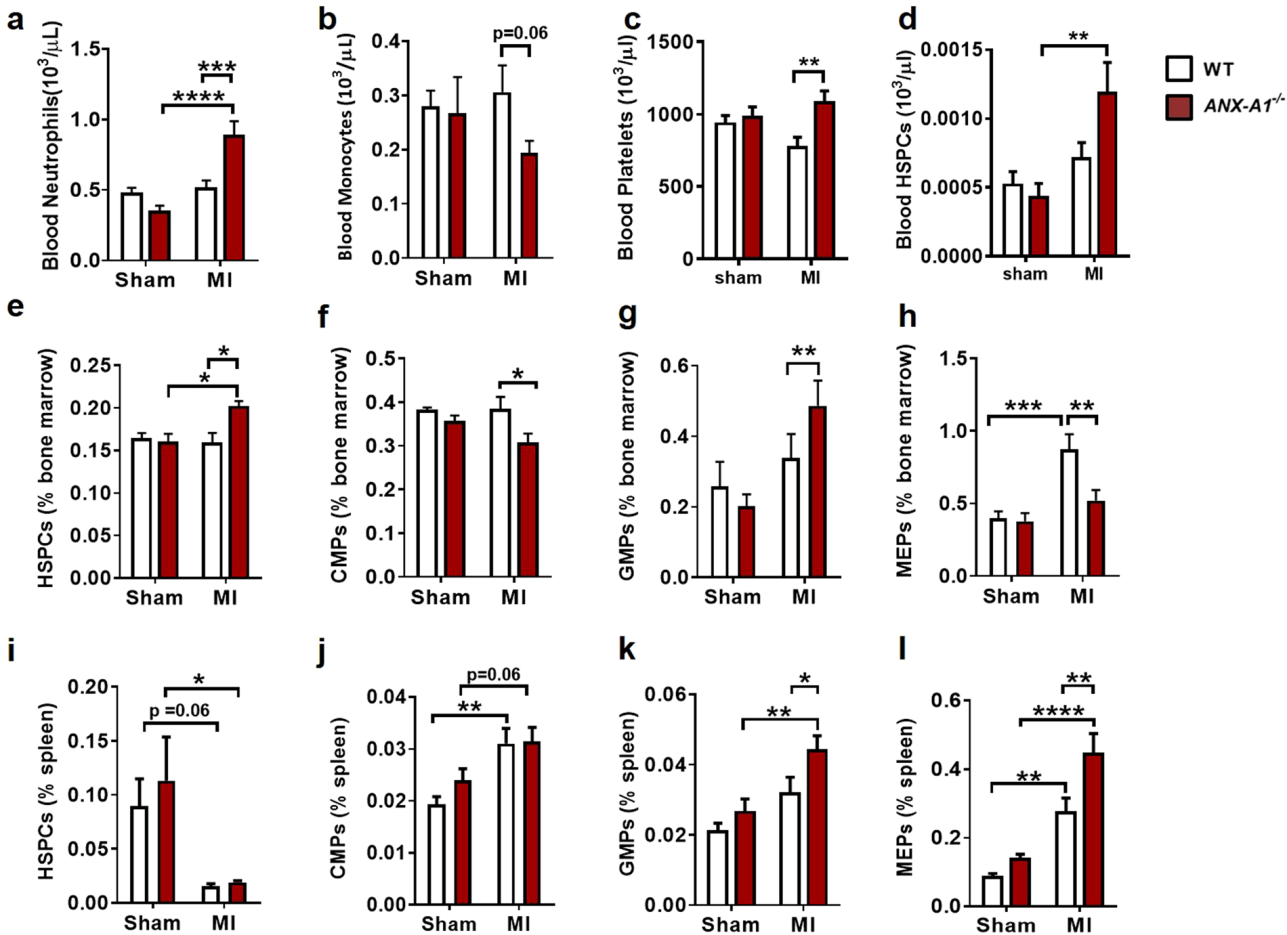
Progenitor cell levels in *ANX-A1*^−/−^ mice 8 days after MI. (**a**–**d**) Flow cytometric analysis of haematopoietic stem and progenitor cells in the blood, (**e**–**h**) bone marrow and (**i**–**l**) spleen in WT (open bars) and *ANX-A1*^−/−^ mice (red-shaded bars). HSPC, haematopoietic stem progenitor cells; CMP, common myeloid progenitor; GMP, granulocyte-macrophage progenitors; MEP, megakaryocyte-erythroid progenitors. HSPCs, lineage-negative (lin^−^), Sca-1^+^, c-Kit^+^ cells. Data is presented as mean ± SEM; *P < 0.05, **P < 0.01, ***P < 0.001, ****P < 0.0001 (Two-way ANOVA followed by Tukey’s post-hoc test). n = 7–9 (shams) and n = 11–12 (MI groups), with WT and *ANX-A1*^−/−^ mice shown in white and red bars, respectively.

To investigate the source driving the enhanced inflammation response in *ANX-A1*^−/−^ mice, we assessed myeloid populations in bone marrow, spleen and blood after 8 days of permanent LAD occlusion. Overall, we observed ANX-A1 deficiency did not impact on myeloid cell numbers in any of these haematopoietic organs in the absence of ischaemic insult (Fig. 4). However, *ANX-A1*^−/−^ mice subjected to MI exhibited an altered pattern of circulating myeloid cells. We observed an increase in blood neutrophil numbers in *ANX-A1*^−/−^, but not in WT mice (Fig. 4a). There was also a trend for lower numbers of circulating blood monocytes in *ANX-A1*^−/−^ mice post-MI (Fig. 4b). This could reflect a persistent inflammatory event in *ANX-A1*^−/−^ mice, with increased monocyte infiltration into the infarcted myocardium. We also observed a significant increase in circulating platelets and HSPC (Fig. 4d) in *ANX-A1*^−/−^ mice post-MI, which might also promote leukocyte recruitment (Fig. 4c). We next examined the source of these circulating myeloid cells, observing an expansion of HSPCs in bone marrow from *ANX-A1*^−/−^ mice post-MI (Fig. 4e), accompanied by a reduction in common myeloid progenitors (CMPs, Fig. 4f) and megakaryocyte-erythroid progenitors (MEPs, Fig. 4h). Interestingly, there was an expansion of granulocyte-macrophage progenitors (GMPs, Fig. 4g), suggesting the bone marrow was likely prioritising the production of neutrophils. In addition, the reduction in MEP numbers in the bone marrow of A*NX-A1*^−/−^ mice post-MI suggests the increased circulating platelets were derived from an alternative source (Fig. 4h). Thus, we next examined if HSPCs had mobilised from the bone marrow into the circulation, promoting extramedullary haematopoiesis. In the spleen we failed to identify more HSPCs (Fig. 4i), however we observed more GMPs and MEPs (Fig. 4k,l). This suggests that the mobilised HSPCs quickly matured into these progenitors, likely also giving rise to circulating neutrophils and platelets.

### ANX-A1 deficiency alters macrophage inflammatory phenotype and function

**Figure 5:**
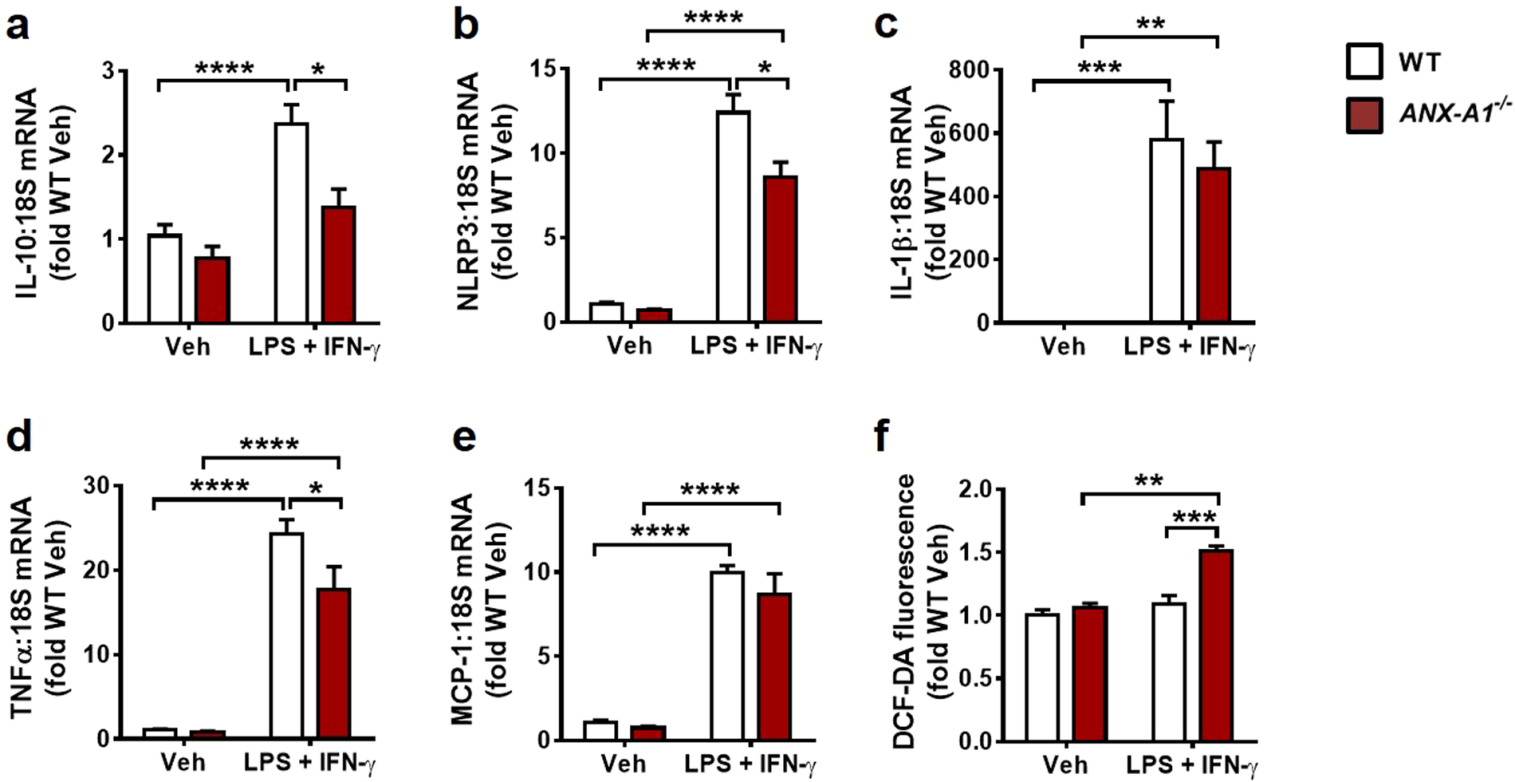
ANX-A1 deficiency alters macrophage inflammatory phenotype and function. Gene expression of (**a**) the anti-inflammatory cytokine IL-10 and pro-inflammatory mediators (**b**) NRLP3, (**c**) IL-1β, (**d**) TNFα and (**e**) MCP-1 in BMDMs derived from WT and *ANX-A1*^−/−^ mice, determined in response to 6 h vehicle or LPS + IFNγ stimulation. n = 5 vehicle and n = 6 LPS + IFNγ-treated WT BMDMs; n = 5 vehicle and n = 4 LPS + IFNγ-treated *ANX-A1*^−/−^ BMDMs, isolated from a total of 3 WT and 3 *ANX-A1*^−/−^ adult male mice. ANX-A1 deficiency significantly impairs responses of IL-10, NRLP3 and TNFα to LPS + IFNγ stimulation. (**f**) ANX-A1 deficiency significantly enhances macrophage ROS levels after 24 h LPS + IFNγ stimulation in BMDMs derived from WT and *ANX-A1*^−/−^ mice, consistent with impaired macrophage function in the context of ANX-A1 deficiency. n = 3 WT and n = 3 *ANX-A1*^−/−^ separate adult male mouse BMDMs, each studied in quadruplicate. Data expressed as fold WT vehicle, presented as mean ± SEM, with WT in white and *ANX-A1*^−/−^ BMDMs in red-shaded bars. *P < 0.05, **P < 0.01, ***P < 0.001, ****P < 0.0001 (Two-way ANOVA followed by Tukey’s post-hoc test).

To investigate the impact of ANX-A1 deficiency on macrophage phenotype and function, we assessed inflammatory and reactive oxygen species (ROS) responses to lipopolysaccharide (LPS)-interferonγ (IFNγ) stimulation in primary bone-marrow derived macrophages (BMDMs) isolated from *ANX-A1*^−/−^ and WT mice (Fig. 5). Overall, we observed that *ANX-A1*^−/−^ mice exhibited an impaired anti-inflammatory response to 6 h LPS + IFNγ stimulation, as evident on gene expression of interleukin-10 (IL-10, Fig. 5a). Intriguingly, ANX-A1 deficiency also blunted pro-inflammatory responses to both nucleotide-binding domain and leucine-rich-repeat protein-3 (NLRP3) and tumour necrosis factor-α (TNFα; Fig. 5b,d), without affecting LPS + IFNγ stimulation of interleukin-1 β (IL-1β) or monocyte chemotactic protein-1 (MCP-1) expression (Fig. 5c,e). However, BMDMs isolated from *ANX-A1*^−/−^ mice exhibited enhanced ROS levels response to 24 h LPS + IFNγ stimulation, detected via dichloro-fluorescein diacetate (DCFDA) detection (Fig. 5f), consistent with impaired macrophage function in the context of ANX-A1 deficiency.

### ANX-A1 deficiency exaggerates cardiac inflammation post-MI *in vivo*

**Figure 6:**
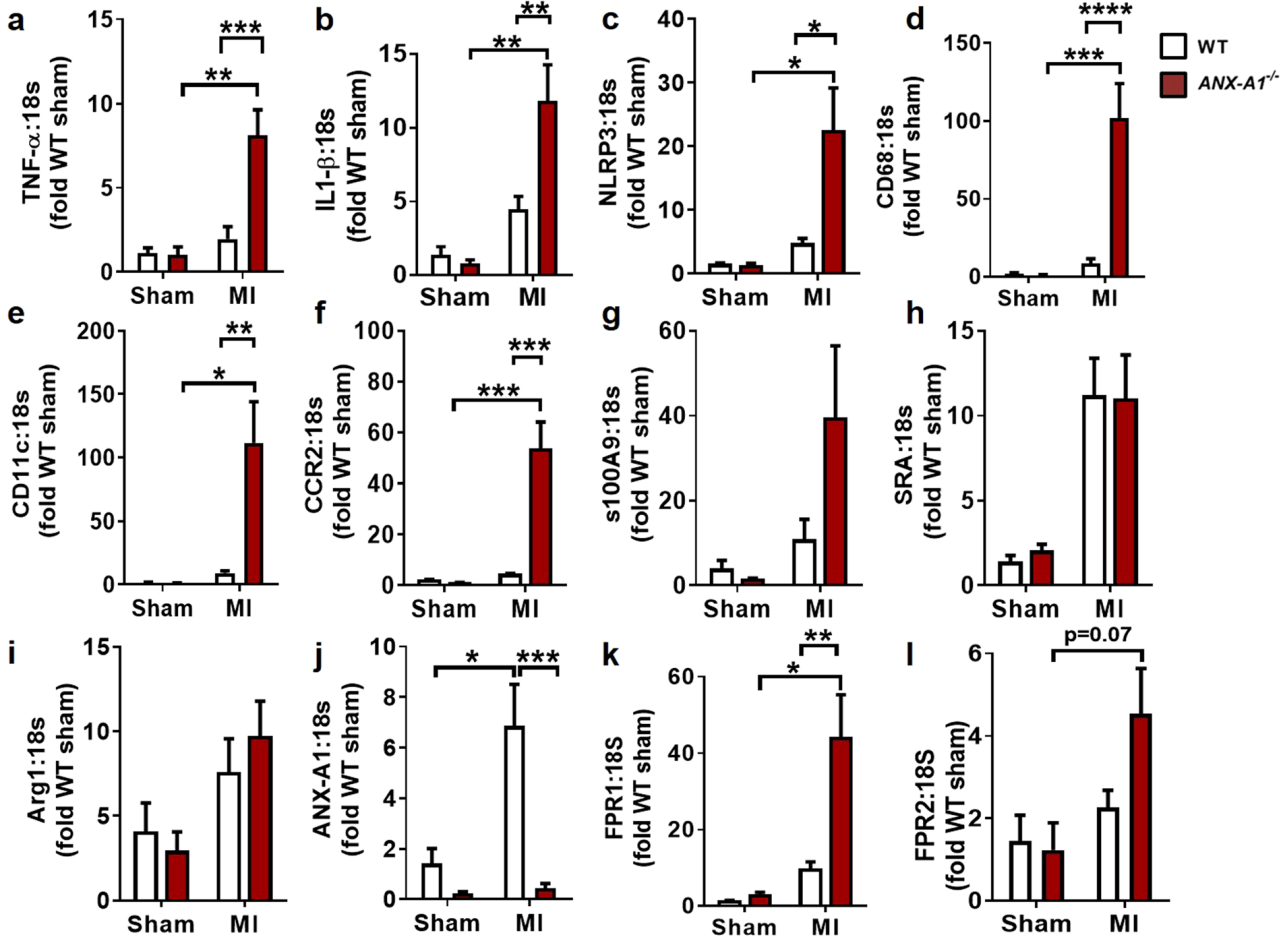
Increased local inflammation in *ANX-A1*^−/−^ mouse myocardial 8 days after MI. LV gene expression of pro-inflammatory cytokines (**a**–**c**) TNF-α, IL-1β and NLRP3, (**d**,**e**) macrophage markers CD68, CD11c, (**f**) the monocyte marker, CCR2, (**g**) M1-macrophage marker S100A9, (h-i) M2-macrophage markers Arg-1 and SR-A, and (j-l) ANX-A1, FPR1 and FPR2 in infarcted LV tissue. TNF-α, tumor necrosis factor-α, IL-1β, interleukin-1β; NLRP3, nucleotide-binding domain and leucine rich repeat protein; Arg, arginase1; SR1, scavenger receptor-1. Data expressed as fold WT sham. WT mice (open bars) and *ANX-A1*^−/−^ mice (red-shaded bars). Data is presented as mean ± SEM, *P < 0.05, **P < 0.01, ***P < 0.001, ****P < 0.0001 (Two-way ANOVA followed by Tukey’s post-hoc test). n = 4 for shams, n = 8–11 for MI groups.

Next, we examined expression of a series of inflammatory related genes including the ANX-A1/FPR system, at 8 days after MI (Cohort 3). The expression of inflammation genes tended to increase in WT mice after MI, but did not reach statistical significance. Interestingly, the expression of inflammatory cytokines, TNF-α and IL-1β (known to promote myelopoeisis and injured tissue monocyte-to-macrophage differentiation), were significantly upregulated ( > 8-fold) in *ANX-A1*^−/−^ compared with WT hearts (Fig. 6a,b). In addition, elevated expression of NLRP3, the regulator of IL-1 β maturation, was also evident in WT mice and exaggerated in *ANX-A1*^−/−^ mice (P < 0.05; Fig. 6c). We also observed significant increases in LV expression of the macrophage markers, CD68 and CD11c, and the monocyte marker CCR2 (by > 50-fold), in *ANX-A1*^−/−^ hearts (Fig. 6d–f). Together, these expression changes suggest that there is enhanced skewing of M1 macrophages towards MI polarisation in *ANX-A1*^−/−^ mice, consistent with the tendency for increased S100A9 expression in *ANX-A1*^−/−^ compared with WT after MI (P = 0.15, Fig. 6g). However, the expression of M2-macrophage markers Arg1 and SRA gene was not different between the two genotypes (Fig. 6h,i). As expected, LV ANX-A1 expression was essentially not detectable in *ANX-A1*^−/−^ mice, confirming their genotype (Fig. 6j). MI induced a significant increase in LV ANX-A1 and FPR1 expression in *ANX-A1*^−/−^ hearts compared with WT (P < 0.01; Fig. 6k). There was also a tendency for increased FPR2 expression (P = 0.07; Fig. 6l), suggesting that upregulation of the cardiac FPR system may occur as a possible compensatory mechanism for the lack of ANX-A1 in these mice.

### ANX-A1 deficiency exacerbates early cardiac remodelling post MI

**Figure 7:**
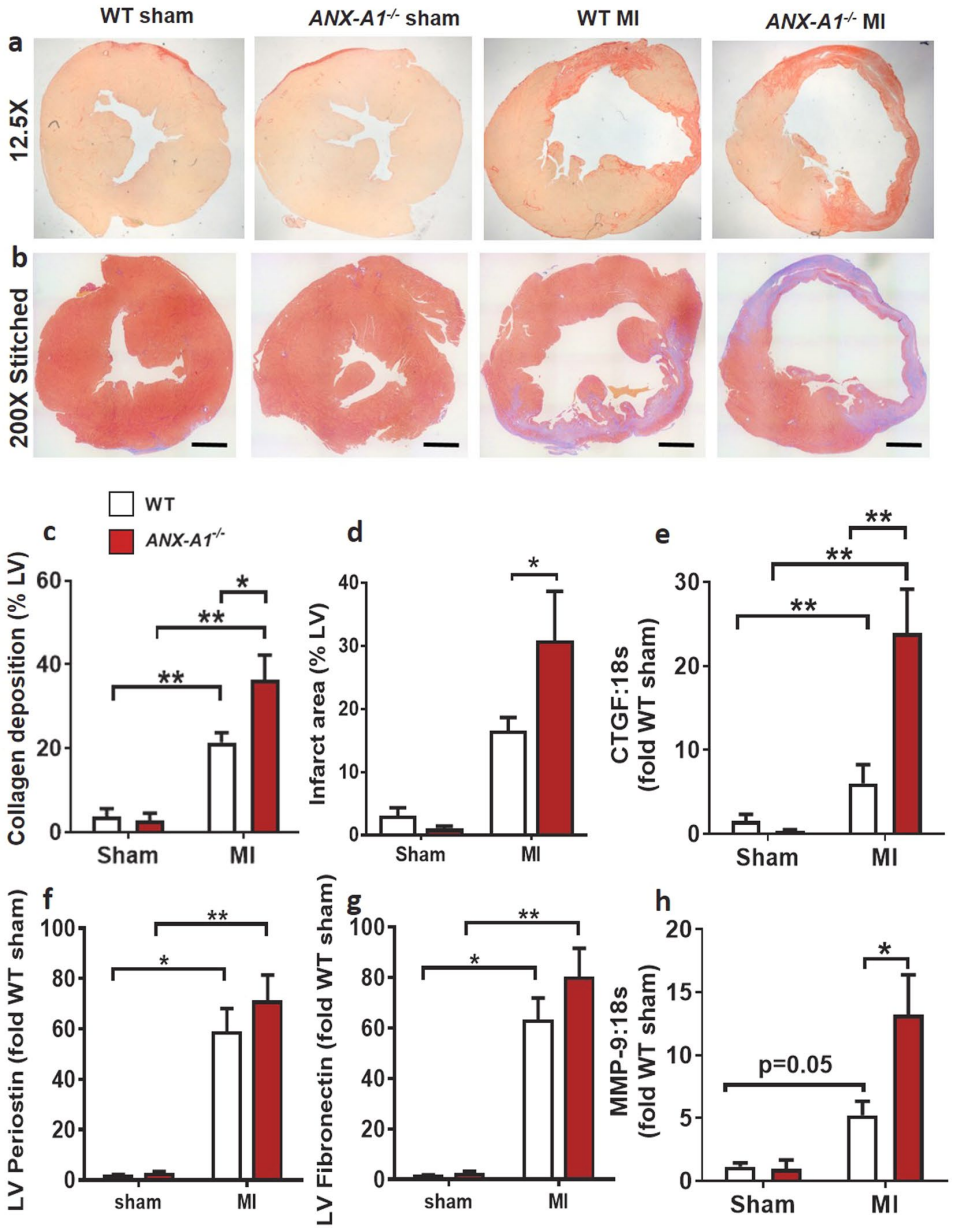
Deficiency of ANX-A1 exacerbates early cardiac fibrosis 8 days after MI. (**a**) Representative images of Sirius red-stained LV sections (scale bar 500 µm), showing increased collagen deposition (pink-red) following MI in *ANX-A1*^−/−^ mice. (**b**) Representative images of Masson’s trichrome stained LV (scale bar 1 µm) each comprised of multiple stitched images. The percentage of blue-stained extracellular matrix of each stitched LV image was assessed relative to the total area. (**c**) Quantification of cardiac collagen content (% of whole LV) in *ANX-A1*^+/+^ *mice* and *ANX-A1*^−/−^ mice. (**d**) Quantification of cardiac infarct area as indicated by blue-stained ECM (% of whole LV) in *ANX-A1*^+/+^ *mice* and *ANX-A1*^−/−^ mice. LV expression of the pro-fibrotic genes (**e**) CTGF, (**f**) periostein, (**g**) fibronectin and (**h**) MMP9. Data is presented as mean ± SEM, expressed as fold change *ANX-A1*^+/+−^ sham. *P < 0.05, **P < 0.01 (Two-way ANOVA followed by Tukey’s post-hoc test for all panels except 7d, where posthoc analysis used Fishers LSD). n = 3–4 shams, n = 8–11 in MI groups, with WT and *ANX-A1*^−/−^ mice shown in white and red bars, respectively.

In the present study, the more severe cardiac injury induced by 8 days of permanent LAD occlusion resulted in significant fibrosis and morphological changes in both WT and *ANX-A1*^−/−^ mice. All mice in the 8 day MI study were included in the survival analysis. All animals survived the first 48 h after surgical MI. There was no statistical difference in survival between genotypes (Supplementary Fig. S3). Compared with sham-operated mice, MI mice exhibited significantly greater collagen deposition on Sirius red-stained LV sections (by > 20-fold), with comparable levels of enhanced extracellular matrix (ECM) deposition evident on Masson’s trichrome staining. The cardiac fibrosis triggered by 8 days MI on both measures was even more pronounced in *ANX-A1*^−/−^ than WT mice (Fig. 7a–d). The increased cardiac fibrosis induced by MI was paralleled by significantly increased expression of the pro-fibrotic genes, connective tissue-derived growth factor (CTGF, Fig. 7e), periostin (Fig. 7f) and fibronectin (Fig. 7g); expression of matrix metalloproteinase (MMP)-9 also tended to increase in WT (P = 0.05, Fig. 7h). The upregulation of both CTGF and MMP were further increased, by 13-fold (P < 0.05, Fig. 7e) and (>10-fold, Fig. 7h), in *ANX-A1*^−/−^ compared with WT mice, respectively.

MI resulted in obvious morphological cardiac changes and significant changes in the weight of several organs (relative to body weight) after 8 days (Supplementary Fig. 4, Supplementary Table S3). Compared with WT mice, *ANX-A1*^−/−^ mice exhibited increased heart and lung weight (P < 0.001 vs WT) together with upregulated hypertrophic gene expression (ANP, by 8-fold) in *ANX-A1*^−/−^ compared with WT mice after MI (Supplementary Fig. 4). These are all early signs suggestive of progression towards cardiac failure, consistent with exaggerated responses to MI in *ANX-A1*^−/−^ mice.

**Figure 8:**
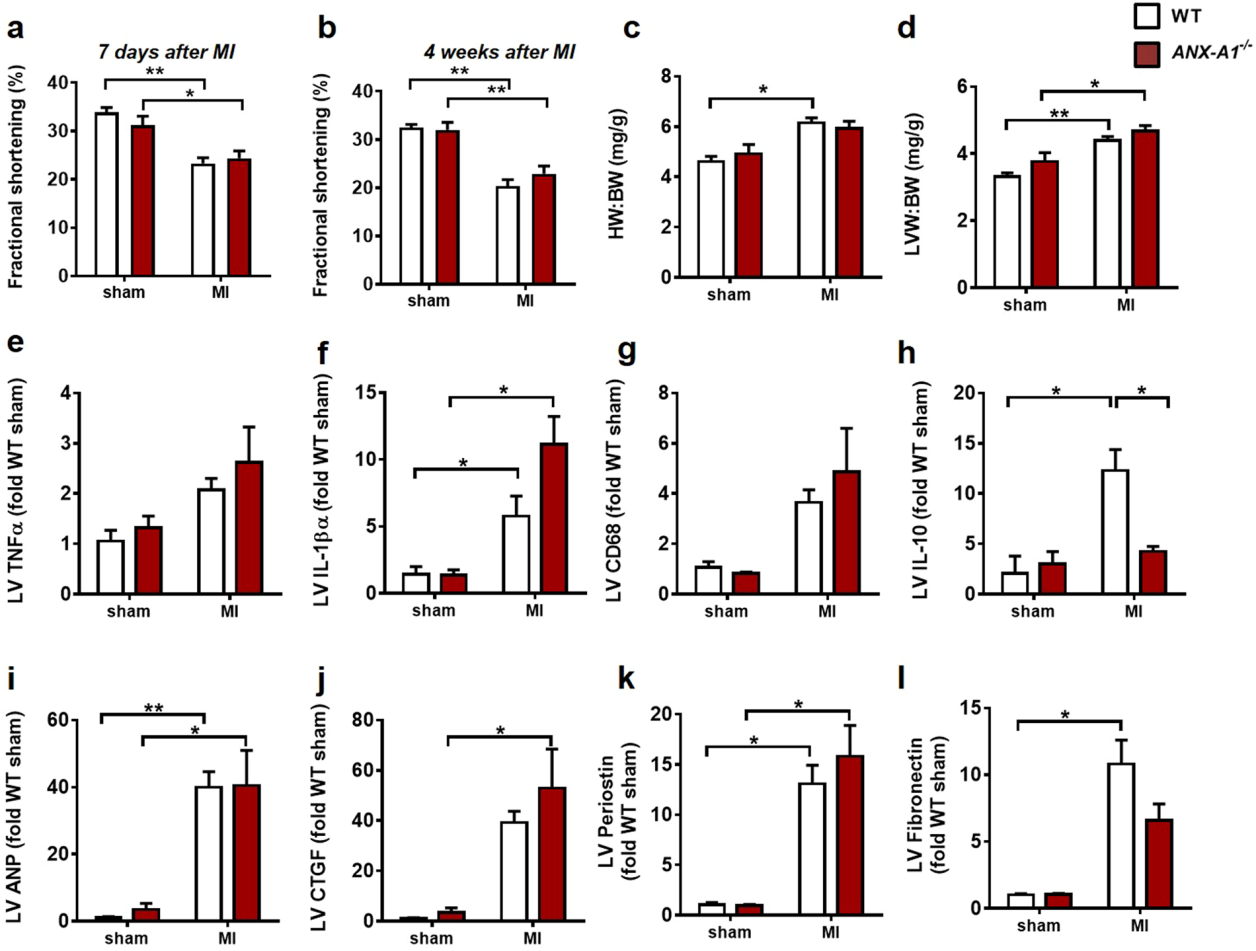
Impact of ANX-A1 deficiency on cardiac response to MI over the longer-term. Cardiac dysfunction, inflammation and remodelling were also assessed after 4 weeks permanent LAD occlusion. MI significantly impaired fractional shortening, as evident after both (**a**) one week and (**b**) 4 weeks after MI. Both (**c**) HW;BW, (**d**) LVW:BW were also increased in response to 4 weeks MI, but were not affected by genotype. Impact on LV gene expression of inflammatory, anti-inflammatory and remodelling markers (**e**) TNFα, (**f**) IL-1β, (**g**) CD68, (**h**) Il-10, (**i**) ANP, (**j**) CTGF, (**k**) perisotin and (**l**) fibronectin are shown. The MI response of pro- vs anti –inflammatory cytokines IL-1β and Il-10 remain elevated 4 weeks after MI, respectively as do the pro-remodelling markers ANP, perisotin and fibronectin in WT mice; only the IL-10 response is significantly blunted in *ANX-A1*^−/−^ compared to WT mice. LV CTGF expression remains significantly elevated in response to MI in *ANX-A1*^−/−^, but not WT, mice. Data is presented as mean ± SEM, expressed as fold change *ANX-A1*^+/+−^ sham. *P < 0.05, **P < 0.01, ***P < 0.001 (Two-way ANOVA followed by Tukey’s post-hoc test). n = 4 shams, n = 9–17 in MI groups, with WT and *ANX-A1*^−/−^ mice shown in white and red bars, respectively.

The impact of a longer-term MI on cardiac dysfunction, inflammation and remodelling was also assessed 4 weeks after permanent LAD occlusion in *ANX-A1*^−/−^ and WT mice. MI significantly impaired fractional shortening (FS); this was evident as early as one week after MI in mice (Fig. 8a) and persists to 4 weeks after MI (Fig. 8b), but was not affected by genotype. In contrast to the early exaggeration of cardiac inflammation and remodelling induced by the deficiency of annexin-A1 after 8 days MI (a phenomenon that drives ventricular dysfunction), by 4 weeks after MI, the majority of these pro-inflammatory and remodelling responses were no longer significantly affected by genotype (Fig. 8c–l). LV expression of the anti-inflammatory cytokine IL-10 was however impaired in response to MI in *ANX-A1*^−/−^ compared to WT mice (Fig. 8h). Further, profibrotic CTGF expression remains significantly elevated in response to MI in *ANX-A1*^−/−^ (but not WT) mice after 4 weeks MI.

## Discussion

With improved survival rates after an initial MI, subsequent HF due to uncontrolled inflammation scarring the myocardium is a significant contributor to mortality in patients with cardiovascular disease. Thus, understanding the importance of endogenous anti-inflammatory pathways that limit myocardial damage is important for development of innovative pharmacotherapies. Using genetically-deficient mice, we now reveal that endogenous ANX-A1, likely acting as an anti-inflammatory and pro-resolving mediator, seems to dampen both inflammation and myocardial damage. The ANX-A1 system may hence represent a novel therapeutic target for ischaemic heart disease, abrogating cardiomyocyte necrosis, inflammation and remodelling *in vivo*, relevant to clinical settings. Interestingly, our result suggest that endogenous ANX-A1 not only suppressed inflammation at the site of the infarct, but also appeared to have a more global effect, by regulating haematopoiesis and macrophage function.

It has been reported that administration of exogenous human recombinant ANX-A1, and its N-terminal derived peptide Ac2-26, limits infarct size after 25 min ischaemia and 2 h reperfusion^11^. In this study, we demonstrated that both the protein and gene expression of endogenous ANX-A1 increase after a myocardial ischaemic insult, and that deficiency in ANX-A1 resulted in exaggerated inflammatory responses. These findings suggest that endogenous ANX-A1 plays an important role in the setting of MI. The cardiac cell type(s) responsible for this upregulation of annexin-A1 are likely multiple; delineating this clearly warrants investigation. We have recently demonstrated that both cardiomyocytes and cardiac fibroblasts express the receptors for annexin-A1, with relative levels of FPR1 > FPR2 > FPR3 in isolated cardiomyocytes, and FPR1 = FPR2 in isolated cardiac fibroblasts; FPR3 are not detected in the latter^20^. Indeed with the increased density of macrophages evident in the present study in the I-R heart *in vivo*, it is likely that cardiac macrophages also become a relevant source of the major FPR subtypes (and perhaps also of their endogenous ligand, annexin-A1), in response to myocardial ischaemic injuries.

In acute MI, the early inflammatory phase is characterised by the accumulation of neutrophils in the ischaemic region as these are the first to infiltrate into the myocardium, followed by macrophages^22^. Accumulation of inflammatory cells in the myocardium has been shown to contribute to myocardial reperfusion injury^7^. It has been postulated that ANX-A1 is released from inflammatory cells, permitting binding to FPR2 at the site of injury^7^. This prevents leukocyte adhesion and infiltration, thus reducing the initial inflammatory response^7^. Previous studies have observed a peak neutrophil accumulation after 24 h after MI^23^. Thus, the 48 h timepoint post I-R utilised here might have been later than the optimal time to assess neutrophil accumulation between the two genotypes^23^. In this study, we observed a significantly higher macrophage density in *ANX-A1*^−/−^ mice, suggesting the inflammatory response to MI is greater in *ANX-A1*^−/−^ mice. Our observation of greater infarct size in *ANX-A1*^−/−^ mice, in conjunction with more marked macrophage infiltration, further emphasis the protective role of ANX-A1 in limiting inflammation-induced myocardial damage. Importantly, we now reveal evidence of the direct consequences of ANX-A1 deficiency on macrophage function and inflammatory phenotype. Macrophage functional response, assessed on LPS-IFNγ-stimulated ROS levels, was exaggerated in *ANX-A1*^−/−^ BMDMs; moreover, anti-inflammatory IL-10 responses were impaired by the deficiency of ANX-A1. As we now show, this impaired anti-inflammatory IL-10 response is also evident in response to MI in the *ANX-A1*^−/−^ myocardium over the longer-term *in vivo*. In addition, our laboratory has previously demonstrated direct cardioprotective roles of ANX-A1 and its derived mimetic peptide Ac2-26 in the absence of inflammatory cells in both rat and mouse myocardium *in vitro*^8,9^, revealing an additional mechanism by which ANX-A1 protects in the myocardium, directly at the level of cardiomyocyte. The increased infarct size in ANX-A1 deficient mice may be in part due to their inability to activate pro-survival kinase pathways, such as Akt, as previously demonstrated^8^, perhaps further exacerbated by altered macrophage function and inflammatory phenotype in this setting.

An ischaemic insult significantly stimulates expansion and mobilisation of HSPCs to increase supply of myeloid cells to the infarcted myocardium^4,24^. We assessed the source of inflammatory cells in the bone marrow and the spleen, testing the hypothesis that haematopoiesis, specifically the myeloid branch, is further expanded and mobilised in *ANX-A1*^−/−^ mice in response to MI. Overall, our results demonstrate that both WT and *ANX-A1*^−/−^ mice subjected to MI exhibited expansion in progenitor populations in the primary (bone marrow) and secondary (spleen) site of haematopoiesis. Interestingly, deficiency in ANX-A1 appeared to favour myeloid skewing, likely producing neutrophils in the bone marrow, together with enhancing HSPC mobilisation to the spleen, where downstream GMPs and MEPs were increased. The precise mechanism responsible for this enhanced egress of HSPCs is unknown, and was beyond the scope of the present study, but may be due to a reduction in the stem cell retention factor CXCL12 in the bone marrow of *ANX-A1*^−/−^ mice^25^, rendering them more susceptible to mobilisation of HSPCs. The expansion of the GMP and MEP populations in the spleen, and the resultant increase in circulating platelets, might also represent an important contributor to enhanced inflammatory profile of the *ANX-A1*^−/−^ mice. Regardless of genotype however, we observed a MI-triggered reduction in splenic HSPCs, which may reflect sympatho-β-adrenoceptor activation as a consequence of acute MI, and/or lymphocyte apoptotic death. Monocytes are triggered in reaction to MI to egress from the spleen towards sites of inflammatory injury to enable them to participate in subsequent healing of the injury^24^, likely as a consequence of increased sympathetic activation^26^. More recently, we further demonstrated that the spleen also releases platelets in response to acute MI; as a result the spleen is rendered partially deplete in both monocytes and platelets following acute MI^27^. Unfortunately neither possibility was sought in the present study, and spleen tissues were not retained (e.g. for determination of markers of sympathetic activation). There was no obvious impact of either MI and/or annexin-A1 deficiency on plasma MCP-1 levels, either after 8 days or 4 weeks MI. Somewhat surprisingly, we detected decreased circulating monocytes in *ANX-A1*^−/−^ compared with WT mice. Even though *ANX-A1*^−/−^ mice exhibited increased macrophage content compared with WT mice, it is likely that *ANX-A1*^−/−^ macrophages are defective in clearing apoptotic cells^28^, resulting in reduced resolution of inflammation in *ANX-A1*^−/−^ mice. Our observations, taken together with the recent suggestion that ANX-A1 acts as a negative regulator of macrophage proliferation^29^, indicate that enhanced myelopoiesis may well contribute to the significant increase in inflammation in *ANX-A1*^−/−^ mice after MI, particularly given that inflammatory myocardial damage (e.g. necroptosis) is widely regarded to exacerbate cardiac damage following MI^7,22,30^.

In this study, we also demonstrated a significant upregulation in macrophage (CD68) and dendritic cells (CD11c) markers of inflammation in the myocardium, associated with pro-inflammatory cytokines TNF-α and IL-1β, in *ANX-A1*^−/−^ mice versus *ANX-A*^+/+−^ mice after 8 days post-MI. These cytokines also enhance activity of MMPs, particularly MMP9, which promotes ECM degradation and aggravates cardiac remodelling as observed in our mice deficient of ANX-A1. Macrophage polarisation results in dual actions of macrophages. In acute MI, the early phase is characterised by a pro-inflammatory macrophages phenotype (M1) and then followed by a later M2 “healing” macrophage phenotype infiltrating into the myocardium. In the present study, deficiency of ANX-A1 resulted in a greater skewing of macrophages to a pro-inflammatory (M1) phenotype, as indicated by upregulated expression of S100A9 gene expression, but no difference in M2 macrophage in both genotypes. Moreover, M1 macrophages may be recruited to injured myocardium as a result of increased MCP-1/CCR2 chemokine/monocyte receptor interactions^22^, consistent with current observations, where further upregulation of LV CCR2 expression in ANX-A1 deficient mice was observed. Our data thus suggests a greater skewing of macrophages towards the pro-inflammatory (M1) phenotype, in the context of ANX-A1 deficiency MI. Where precisely these immune cells in the heart arise and how these are affected by MI and/or ANX-A1 deficiency thus warrants further consideration.

The inflammatory response following MI is critical for infarct healing and scar formation^31^. However, excess inflammation can lead to adverse ventricular remodelling, including fibrosis, LV hypertrophy, and cardiac rupture. Eight days post-MI, LV CTGF expression was markedly increased (13-fold) in *ANX-A1*^−/−^ versus WT mice. This was accompanied by significantly increased myocardial fibrosis. Our study demonstrated for the first time that ANX-A1 deficiency exacerbates myocardial fibrosis post-MI, consistent with a previous study on pulmonary fibrosis^32^. Myocardial morphology changes as an adaptive process to LV remodelling following MI^33^. We observed significant upregulation of the hypertrophic marker, ANP, post-MI, associated with increased heart weight in *ANX-A1*^−/−^ mice, together with extensive collagen deposition, indicating maladaptive LV remodelling. In addition, the increased lung weight in *ANX-A1*^−/−^ mice is an early sign of heart failure, consistent with an exaggerated response in these mice. It is thus likely that deficiency of ANX-A1 accelerates the onset of cardiac ischaemic remodelling. Our selection of the 8 day timepoint enabled us to detect this earlier onset of cardiac remodelling in ANX-A1^−/−^ mice. A component of this early remodelling response, particularly the increased HW:BW and LV ANP expression are however not evident in WT mice until 4 weeks after MI (Fig. 8). Importantly in mice, fibrotic signalling commences from day 3 onwards and by day-8, regional collagen deposition is already very evident (as our data for both Sirius red and Masson’s trichrome clearly indicate). The day-8 timepoint represents the early phase of fibrotic healing that is closely coupled with the preceding inflammatory process and is a critical determinant for ventricular remodelling, as the heart further progresses towards chronic heart failure.

### Limitations of the Study

In the present study, we demonstrated that global deficiency of ANX-A1 exhibits increased early cardiac remodelling and inflammatory responses to myocardial ischaemic insults in mice. Whilst this was accompanied by an altered pattern of HSPC expansion and mobilization after 8 days of MI, we did not specifically dissect out whether the mechanism(s) responsible for the exaggerated myocardial injury evident in *ANX-A1*^−/−^ mice was attributed to disruption of ANX-A1 in cardiomyocytes, macrophages, fibroblasts and/or cardiac endothelial cells. The specific role of HSPC mobilisation in the cardiac consequences of ANX-A1 deficiency was not sought, nor was the ability of supplementation with ANX-A1 (or indeed a broader anti-inflammatory intervention) to rescue the exaggerated early cardiac response to MI in the absence of ANX-A1. Nonetheless, as we have previously demonstrated, exogenous treatment with ANX-A1 peptide mimetic confers direct protective effects on cardiomyocytes *in vitro* (in the absence of circulating inflammatory cells)^8,9^. Our new evidence here now affirms that deficiency of ANX-A1 increases susceptibility to myocardial ischaemic injury, consistent with the view that endogenous ANX-A1 is a negative regulator of cardiac pathological responses. A final limitation of the current study is that myocardial function was not assessed in the early response to myocardial I-R injury, but only the later response to permanent LAD ligation. However, our new evidence here now affirms that deficiency of ANX-A1 increases susceptibility to myocardial ischaemic injury, consistent with the view that endogenous ANX-A1 is a negative regulator of early cardiac pathological responses (particularly in the first week post insult).

## Conclusions

*ANX-A1*^−/−^ mice exhibited increased LV necrosis, inflammation and adverse remodelling compared with WT mice after a myocardial ischaemic insult. Endogenous anti-inflammatory responses are also likely impaired (based on the impact of ANX-A1 deficiency on IL-10 responses in both isolated macrophages and the intact myocardium over the longer-term). This may be due to a greater expansion of HSPCs, and their altered pattern of mobilisation, in *ANX-A1*^−/−^ mice. These findings suggest that endogenous ANX-A1 is cardioprotective *in vivo* not only through reducing infiltration of inflammatory cells into injured myocardium, but also reducing the origins of inflammatory cells, especially the myeloid branch. ANX-A1-based strategies may hence provide additional benefits over traditional anti-inflammatory strategies for enhancing myocardial viability and limiting adverse cardiac remodelling, particularly in the first few days after an ischaemic insult; such strategies may hence represent novel therapy for the early treatment of acute MI in clinical settings.

## Methods

### Animals and materials

All animal research was conducted in accordance with the National Health and Medical Research Council of Australia guidelines, and Directive 2010/63/EU of the European Parliament in the protection of animals used for scientific purpose. Animal care and experimental protocols were approved by the Alfred Medical Research Education Precinct (AMREP) Animal Ethics Committee. Adult male Sprague Dawley rat, and wild type (WT) and *ANX-A1*^−/−^ mice (on a C57BL/6 background) were bred and housed in the AMREP Animal Centre and maintained under a 12 h light/dark cycle. All reagents were purchased from Sigma-Aldrich (St. Louis, USA) except where indicated, and were of analytical grade or higher.

### Genotyping

*ANX-A1*^−/−^ mice, generated using a dual-purpose targeting vector designed to simultaneously inactivate the gene and promoter for *ANX-A1*^34^, were kindly provided by Prof Rod Flower, William Harvey Research Institute, London UK. Genotyping was performed to confirm absence of the *ANX-A1*^−/−^ gene using polymerase chain reaction (PCR). Briefly, DNA was extracted from mouse tail clips, followed by PCR with forward and reverse primers for *ANX-A1* allele sequence for WT and Lac Z allele sequence (the null sequence) for *ANX-A1*^−/−^ as described previously^34^. Samples were run on a 2% agarose gel containing ethidium bromide and imaged using an UV Illuminator. 700 bp and 220 bp band products were determined for WT and *ANX-A1*^−/−^ (Lac-Z) respectively (See Supplementary Fig. S1).

### Expression of the ANX-A1 system after cardiac ischaemic injury *in vitro* and *in vivo*

To assess the influence of an ischaemic insult on the expression of ANX-A1 and its receptors (FPR1 and FPR2), firstly we examined protein levels of ANX-A1 in rodent hearts subjected to ischaemia and reperfusion in a Langendorff model as described previously^9^. Briefly, after thoracotomy, the heart was removed from an anaesthetised (100 mg/kg ketamine, 12 mg/kg xylazine, I.P.) rat hearts and mounted onto a Langendorff perfusion apparatus and buffer-perfused at constant flow (10 mL/min) at 37 °C. Following 20 min equilibration, hearts were subjected to global no-flow ischaemia for 30 min, followed by restoration of flow for a further 30 min, conditions previously shown to limit recovery of LV function to approximately 60% of pre-ischaemic levels^8,35^. Sham (normoxic) hearts were perfused for a total of 80 min. At the end of reperfusion, hearts were then snap-frozen and stored at −80 °C until biochemical analysis. To detect the protein expression of ANX-A1 (both 37 kDa and 34 kDa isoforms), LV (~30 mg) were homogenised and protein extracts (100 µg/per lane) were loaded 10% SDS-PAGE for electrophoresis as described previously^10,36^. Blots were probed with anti-ANX-A1 (1:1000, Cell Signaling-3299) followed by the loading control, anti-β-actin (1:1000, Cell Signaling-4967). We then examined the influence of an ischaemic insult on gene expression of ANX-A1 and FPRs in mice *in vivo*, as described previously^20^. Quantitative-real time PCR was conducted using SYBR green chemistry (Life Technologies, Victoria, Australia) to measure the expression of the FPR system (ANX-A1, FPR1, FPR2), using the Applied Biosystems ABI Prism 7700 Sequence Detection System. Ribosomal 18 S was used as the endogenous control. Primers were generated from murine sequences (Supplementary Table S1) published on GenBank and synthesised by GeneWorks (Hindmarsh, Australia). Gene expression levels were calculated using the 2^−ΔΔct^ method to detect relative fold differences^20^.

### Surgery to induce myocardial I-R and MI *in vivo*

Adult male *ANX-A1*^−/−^ or WT C57BL/6 mice were randomly assigned to either myocardial I-R injury, MI or sham *in vivo*. Briefly, mice were anaesthetised, (ketamine 80 kg/mg, xylazine 30 mg/kg and atropine 1.2 mg/kg, I.P.), mechanically-ventilated and left thoracotomy performed to expose the left anterior descending (LAD) coronary artery. For cohorts 1 and 2, the LAD was ligated using 7.0 silk suture with a slip knot enclosing two releasing rings. Regional ischaemia was induced for 1 h, then blood flow through the LAD was re-established by releasing the slip-knot, as we have described previously^20,21^. Mice in cohorts 1 and 2 were then subjected to reperfusion for 24 h and 48 h respectively, which are optimal time points for the assessment of cardiac necrosis and early cardiac inflammation *in vivo*^20^. Animals in cohorts 3 and 4 were subjected to a more severe ischaemic insult, permanent LAD ligation for 8 days (cohort 3) or 4 weeks (cohort 4)^20,21^, to assess the mobilisation of HSPCs, severity of regional inflammation and cardiac remodelling. Sham animals underwent identical surgical procedures, but without ligation. At study end, all mice were euthanised, heparinised blood was collected by cardiac puncture, and the lungs and heart collected. Atria, right ventricle (RV) and LV were separated and weighed.

### Determination of cardiac necrosis at early I-R injury *in vivo*

Cardiac necrosis was assessed in cohort 1 mice after 24 h reperfusion, the optimal time point of cardiac necrosis after an ischaemic insult. Plasma levels of cTnI were determined at this timepoint using a commercially available high-sensitivity mouse cTnI ELISA kit (Life Diagnostic Inc., Pennsylvania, USA) as per the manufacturer’s instructions^20,21^. Infarct size (IS) in relation to the area-at-risk (AAR) was also determined, using standard 2,3,5-triphenyltetrazolium chloride (TTC) staining. Briefly, the LAD was tightly re-occluded in anaesthetised mice after 24 h reperfusion, and Evans blue dye (0.1 mL, 5%) was injected into LV. The heart was then excised and rinsed in cold saline to remove excess dye. The LV was isolated, frozen at −20 °C and then cut transversely into six to seven slices of 1.0 mm thick. LV slices were incubated for 45 min with 1.5% TTC solution at 37 °C. The presence of Evans blue indicated normal tissue perfusion, whereas the absence of dye infiltration indicated lack of perfusion to that region. Moreover, brick red areas indicated viable myocardium, whereas white or yellowish regions demarcated necrotic tissue. The slices were mounted between glass slides, and images were acquired digitally using a surgical microscope (Leica Wild M3B, Heerbrugg, Switzerland) coupled with a digital camera (Nikon Cool-PIX4500, Tokyo, Japan). The images were analysed using Image J (Version 1.45 S, National Institute of Health, USA). The non-ischaemic zone (blue area), area-at-risk (AAR) zone (red and white or yellow areas), infarct zone (white or yellow areas) and total LV were outlined and quantified with the investigator blinded to sample identity. IS was calculated as the percentage of the infarct zone in the AAR^20,21^.

### Assessment of early cardiac inflammation following I-R injury *in vivo*

The LV from mice in Cohort 2 subjected to 1 h ischaemia and 48 h reperfusion were processed as fresh-frozen in Tissue-Tek® optimal cutting temperature (OCT) compound (Tissue-Tek, Torrance, USA) and cut into 6 μm for immunofluorescent detection of cardiac macrophage and neutrophil density, as we have described^20^. Briefly, LV sections were then incubated with CD68 + primary antibody for macrophage detection, or Ly-6b.2 primary antibody for neutrophil detection (1:200, ABD Serotec, Raleigh, USA) for 1 h, followed by 30 min incubation with an Alexa Fluor 546 secondary antibody (1:200, Invitrogen, Carlsbad, USA). Finally, sections were incubated with 0.001% Hoechst 33342 (Invitrogen, Melbourne, Australia) for 30 min, to elicit nuclear staining as described previously^20^. Macrophage and neutrophil stained sections were counted manually as CD68^+^ and Ly-6b.2 overlay with DAPI, with the investigator blinded to sample identity. Numbers of CD68^+^ positive cells in 10 representative squares per LV were averaged, and calculated per square millimeter^23^.

### Assessment of mobilisation of haematopoietic stem cells following MI *in vivo*

Haematopoietic stem cells from the bone marrow, spleen and blood were isolated as previously described^37^. HSPCs were identified as lineage negative (lin^neg^), cKit^hi^ and Sca1^hi^. Haematopoietic progenitor cells (HPCs) were identified as lin^neg^ CD117^hi^ and Sca-1^lo^. Progenitor cells were then further differentiated by expression of CD16/CD32 (FcγRII/III). Granulocyte-macrophage progenitors (GMP) were identified as lin^neg^ CD117^hi^ Sca-1^lo^ and FcγRII/III^hi^; megakaryocyte-erythroid progenitors (MEP) were identified as lin^neg^ CD117^hi^ Sca-1^lo^ and FcγRII/III^lo^. Monocytes were identified as CD45^hi^ CD115^hi^ and neutrophils as CD45^hi^ CD115^lo^ and Ly6-C/G^hi^.

### Isolation and culture of murine bone marrow-derived macrophages

BMDMs were isolated from untreated WT and *ANX-A1*^−/−^ mice, as previously described^38^. Following euthanasia, femurs and tibias were dissected from mice, and bone marrow cells flushed with supplemented RPMI + Glutamax media (20% L-cell media containing macrophage colony-stimulating factor to induce differentiation, 15% fetal bovine serum and 1% penicillin streptomycin). Red blood cells were eliminated after incubation in Ammonium-Chloride-Potassium (ACK) lysis buffer for 2 min. After quenching cells in excess media and centrifugation, cells were resuspended in media and seeded into 150 mm tissue culture plates. Media was replaced on day 2. On day 4, cells were detached from plates using cell scrapers and split at a 1:2 ratio. After a further 8 days, cells were counted, and 10,000 cells seeded onto clear-bottomed 96-well plates for the DCFDA ROS detection assay, and 500,000 cells seeded into a 12-well plate for RT-PCR analysis. Following overnight incubation, cells were then stimulated with LPS (100ng/µl) and IFN-γ (20ng/µl) for 6 or 24 hours, for RT-PCR and DCFDA assay, respectively. For RT-PCR analysis, cells were washed with PBS and cells scraped and collected in Trizol for subsequent RNA extraction.

### RNA extraction and gene expression

Briefly, RNA was extracted from BMDMs or LV tissues using TRIzol® (Invitrogen, Life Technologies, Mulgrave, VIC, Australia) as previously described^36^. Taqman reverse-transcription reagents (Applied Biosystems, Mulgrave, VIC, Australia) were used to generate approximately 20ng/µl cDNA from 1 µg of DNase-treated RNA via transcription. Quantitative-real time PCR was conducted using SYBR green chemistry (Life Technologies, Victoria, Australia) to measure the expression of inflammatory genes (TNF-α; IL-1β; NLRP3; MCP-1 [also known as CCL2] and IL-10) using the Applied Biosystems ABI Prism 7700 Sequence Detection System described previously^20^ using sequences in Supplementary Table S1.

### DCFDA Assay to determine ROS levels

After 24 hours of LPS/IFN-γ stimulation, WT and ANX-A1^−/−^ BMDMs were incubated in phosphate buffered saline (PBS; with Ca^2+^/Mg^2+^) containing DCFDA (5 μM), an intracellular probe to detect ROS for 40 min at 37 °C^39^. All treatments were studied in quadruplicate. Cells were subsequently washed with PBS prior to incubation in either PBS alone (control) or LPS (100ng/µl)/IFN-γ (20ng/µl), and fluorescence quantified after 90 minutes on the Omega Fluorstar reader (485 nm excitation and 530 nm emission).

### Assessment of cardiac inflammation following severe ischaemic injury *in vivo*

The LV was collected from anaesthetised mice in Cohorts 3 (following 8 days of permanent LAD ligation to induce MI) and 4 (4 weeks of permanent LAD ligation) and were dissected, blotted dry, and weighed. The infarcted area from cohort 3 mice was separated and snap-frozen for gene expression analysis. Hearts from cohort 4 mice were pinned out and photographed prior to snap-freezing for gene expression analysis. RNA was extracted from infarcted LV using TRIzol®, prior to reverse-transcription and real time PCR as described above. The expression of inflammatory genes (TNF-α, IL-1β, NLRP3, CD68, CD11c, C-C chemokine receptor type 1 [CCR2]), as well as M1 macrophage marker [S100A9], M2 macrophage marker (macrophage scavenger receptor, SRA; Arginase1, Arg1), the FPR system (ANX-A1, FPR1, FPR2), fibrotic genes (connective tissue growth factor CTGF, periostin, fibronectin, MMP-9), and a hypertrophic gene (atrial natriuretic peptide, ANP), using the Applied Biosystems ABI Prism 7700 Sequence Detection System^20^, using sequences in Supplementary Table S1. Plasma levels of MCP-1 were also determined after 8 days and 4 weeks permanent LAD occlusion (cohorts 3 and 4), using a commercially available, high-sensitivity mouse MCP-1 ELISA Kit (RayBiotech Inc., Georgia, USA), as per the manufacturer’s instructions.

### Assessment of cardiac remodeling and function *in vivo*

LV tissues collected from mice in Cohort 3, after 8 days permanent LAD occlusion, were fixed in neutral buffered formalin, embedded in paraffin by the Alfred Pathology Service (Melbourne, Australia) and sectioned at 4 mm with a Leica 2135 microtome (Leica Microsystems, Wetzlar, Germany). Sections were stained with picrosirius red (0.1%, Fluka, Bucks, Switzerland; pH 2) for assessment of cardiac collagen deposition^20,36,40^. The area of picrosirius red staining (% LV area) was quantitatively measured using Image-Pro Plus software (Media Cybernetic Inc.) as previously described^20,36,40^. ECM of separate paraffin-fixed cardiac sections were stained using Massons’ Trichrome kit (AMT.K) from Australian Biostain P/L (Traralgon, Victoria, Australia). The percentage of blue stained ECM to total heart area was quantified by assessing stitched images of a cross-section of each heart using ImageJ software (v1.48a National Institutes of Health USA). Haematoxylin-eosin (H&E)-stained cardiac sections were photographed under light microscopy, also as previously described^40^. M-mode echocardiography was also performed in anaesthetised mice allocated to Cohort 4 (ketamine/xylazine/atropine: 60/6/0.7 mg/kg I.P.) just prior to tissue collection, to assess fractional shortening after 1 and 4 weeks post MI, utilising a Philips iE33 ultrasound machine (North Ryde, NSW, Australia) with a 15 MHz linear (M-mode) transducer as previously described^20,40,41^ with ProSolv® Cardiovascular Analyzer 3.5 (Problem Solving Concepts, Inc, Indiana, USA). All morphometric and echocardiographic analyses were performed with the investigator blinded to sample identity.

### Assessment of cardiac rupture after MI *in vivo*

Causes of premature death prior to study endpoint were determined by autopsy examination of all animals found dead in the MI study (cohorts 3 and 4, permanent LAD occlusion), as previously described^42^. Cardiac rupture was confirmed by the presence of a blood clot around the entire heart and chest, and a perforation or a tear of the infarcted wall. The number of animal deaths was used to generate a Kaplan-Meier survival curve^42^.

### Statistical Analysis

GraphPad Prism software (Version 6.00, La Jolla, California, USA) was used to perform statistical analysis. Data was expressed as mean ± SEM. An unpaired Student’s t-test was used to analyse ANX-A1 protein expression, IS and AAR. Two-way ANOVA was used for comparing genotype (*ANX-A1*^−/−^ vs WT) and disease (sham vs MI) followed by *post-hoc* analysis as indicated. Multiple comparisons were analysed using Tukey’s Post-hoc. The Kaplan-Meier survival curve was analysed by the log-rank (Mantel-Cox) test. A value of P < 0.05 was considered significant.

### Data Availability

The data that supports the findings of this study are available from the corresponding authors on request.

## Electronic supplementary material

Supplementary
