# Author Correction: Endogenous Annexin-A1 Regulates Haematopoietic Stem Cell Mobilisation and Inflammatory Response Post Myocardial Infarction in Mice *In Vivo*

**DOI:** 10.1038/s41598-018-24994-9

**Published:** 2018-05-02

**Authors:** Cheng Xue Qin, Siobhan B. Finlayson, Annas Al-Sharea, Mitchel Tate, Miles J. De Blasio, Minh Deo, Sarah Rosli, Darnel Prakoso, Colleen J. Thomas, Helen Kiriazis, Eleanor Gould, Yuan H. Yang, Eric F. Morand, Mauro Perretti, Andrew J. Murphy, Xiao-Jun Du, Xiao-Ming Gao, Rebecca H. Ritchie

**Affiliations:** 1 Baker Heart & Diabetes Institute, Melbourne, 3004 Australia; 2 Dept of Pharmacology and Therapeutics, University of Melbourne, Parkville, 3010 Australia; 3 Dept of Physiology, Anatomy and Microbiology, La Trobe University, Bundoora, 3086 Australia; 4 Dept of Medicine, Central Clinical School, Monash University, Melbourne, 3004 Australia; 5 School of Biosciences, University of Melbourne, Parkville, 3010 Australia; 6 Centre for Inflammatory Diseases, Monash University, Clayton, 3168 VIC Australia; 7 Department of Immunology, Monash University, Melbourne, 3004 VIC Australia; 8 William Harvey Research Institute, Barts and The London School of Medicine, Queen Mary University of London, London, United Kingdom

Correction to: *Scientific Reports* 10.1038/s41598-017-16317-1, published online 30 Nobemver 2017

The original version of this Article contained a typographical error in the spelling of the author Annas Al-Sharea, which was incorrectly given as Annas AI-Sharea. This has now been corrected in the PDF and HTML versions of the Article.

