# Supplementary material for: Endogenous Annexin-A1 Regulates Haematopoietic Stem Cell Mobilisation and Inflammatory Response Post Myocardial Infarction in Mice In Vivo
Source: Sci Rep. 2017 Nov 30;7:16615. doi: 10.1038/s41598-017-16317-1 (PMC5709412; doi:10.1038/s41598-017-16317-1)
Supplement: Supplementary file 1 — Supplementary [file 41598_2017_16317_MOESM1_ESM.pdf]

SUPPLEMENTARY RESULTS

ENDOGENOUS ANNEXIN-A1 REGULATES HAEMATOPOIETIC STEM CELL MOBILISATION AND INFLAMMATORY RESPONSE POST MYOCARDIAL INFARCTION IN MICE *IN VIVO*

Running title: Annexin-A1 deficiency exaggerates MI

Cheng Xue Qin[^1,2], Siobhan B Finlayson[1,3,4], Annas Al-Sharea[1,4], Mitchel Tate[1], Miles J De Blasio[1,5], Minh Deo[1], Sarah Rosli[1,6], Darnel Prakoso[1,5], Colleen J Thomas[3], Helen Kiriazis[1], Eleanor Gould[1], Yuan H Yang[6], Eric F Morand[6], Mauro Perretti[8] , Andrew J Murphy[1,7], Xiao-Jun Du[1,4], Xiao-Ming Gao[1#], Rebecca H Ritchie[1,2,4#^].

[1]Baker Heart & Diabetes Institute, Melbourne 3004; [2]Dept of Pharmacology and Therapeutics, University of Melbourne, Parkville 3010, [3]Dept of Physiology, Anatomy and Microbiology, La Trobe University, Bundoora 3086; [4]Dept of Medicine, Central Clinical School, Monash University, Melbourne 3004, [5]School of Biosciences, University of Melbourne, Parkville 3010, [6]Centre for Inflammatory Diseases, Monash University, Clayton 3168, VIC, Australia. [7]Department of Immunology, Monash University, Melbourne 3004, VIC, Australia. [8]William Harvey Research Institute, Barts and The London School of Medicine, Queen Mary University of London, London, United Kingdom

[#]XMG, RHR are joint senior authors. ^CXQ, RHR are joint corresponding authors.

CORRESPONDENCE:

Dr Cheng Xue Qin, PhD, Heart Failure Pharmacology, Baker Heart & Diabetes Institute, 75 Commercial Rd, Melbourne 3004 Australia. Adjunct Research Fellow (University of Melbourne). Phone+613-8532-1374; Fax+613-8532-1100; Email chengxuehelena.qin@baker.edu.au

Prof Rebecca H. Ritchie, PhD, FCSANZ, FAHA; NHMRC Senior Research Fellow, Head Heart Failure Pharmacology, Baker Heart & Diabetes Institute, 75 Commercial Rd, Melbourne 3004 Australia. Phone+613-8532-1392; Fax+613-8532-1100; Email rebecca.ritchie@baker.edu.au

Supplementary Results

Post-mortem characteristics

There were no significant differences between WT and *ANX-A1*$^{-/-}$ mice in body weight, or whole heart, RV, or atrial weights, in terms of absolute weight or when normalised to body weight at both 24h or 48h after I-R (Supplementary Table S2). However, there was a significant increase in post-surgery body weight in *ANX-A1*$^{-/-}$ mice 48h after I-R. In spite of this, the net change in body weight was not statistically different between experimental groups. Likewise, 48h after I-R, heart weight, RV and atria weights, when normalized to body weight, were not significantly different across experimental groups. Interestingly, I-R induced a marked increase in LV weight in *ANX-A1*$^{-/-}$ (but not WT) mice (Supplementary Table S2). In cohort 3, MI induced more significant changes in organ weights relative to body weight 8 days after surgery (Supplementary Table S3). There were no significant differences in body weight, spleen, kidney or RV weight relative to BW observed between all 4 experimental groups in Cohort 4.

Systemic inflammation

Total circulating inflammatory cells was determined by differential cell counts in all four cohorts. There was no significant difference between sham-operated and mice subjected to I-R, in respect to white blood cells (WBC), neutrophils, monocytes, eosinophils and basophils. In addition, there were no genotypes differences (Supplementary Table S2). The 8 days permanent occlusion model elicited more significant changes in systemic circulation, but there was no differences in circulating lymphocytes and white blood cells between genotypes in either sham or MI groups (Supplementary Table S3). Four weeks after permanent occlusion no significant changes in in circulating leukocytes were observed in either genotype in either sham or MI groups (Supplementary Table S3). Plasma MCP-1 levels were determined at study endpoint, 8 days and 4 weeks permanent LAD ligation, in cohorts 3 and 4, as a potential estimate of chemokine-mediated release of splenic monocyte release. There was however no

obvious impact of either MI and/or annexin-A1 deficiency on plasma MCP-1 levels, either after 8 days or 4 weeks MI (albeit only small numbers were studied).

SUPPLEMENTARY FIGURE LEGENDS

Supplementary Figure 1. Genotyping of tail clip DNA by PCR. Lane 1: molecule weight markers; lane 2 and 3, WT mice tail DNA; Lane 4 and 5: *ANX-A1*$^{-/-}$ mice tail DNA. Note 700bp and 220bp band sizes for predicted band for the *ANX-A1* and *LacZ* genes .DNA bands were acquired under 345nm UV light.

Supplementary Figure 2. Impact of deficiency of ANX-A1 on circulating MCP-1 levels after MI. (a) Plasma MCP-1 in mice from Cohort 3, after 8 days MI. (b) Plasma MCP-1 in mice from Cohort 4, after 4 weeks MI. Data is presented as mean ± SEM. P=NS (Two-way ANOVA followed by Tukey's post-hoc test). n= 3 shams, n= 6 in MI groups.

Supplementary Figure 3. Impact of deficiency of ANX-A1 on survival after MI. Day 0 represents surgery day. Peak cardiac rupture tended to occur between days 4-5 post MI. (a) Cohort 3, after 8 days MI. (b) Cohort 4, after 4 weeks MI. Log rank Mantel-COX comparison test (p=NS between genotypes). MI, myocardial infarction.

Supplementary Figure 4. Deficiency of ANX-A1 exacerbates cardiac remodelling 8 days after MI. (a) Representative images of Sirius red-stained LV sections at 200X, showing density of collagen deposition within the infarct (pink). (b) Representative images of H&E-stained LV sections (top row, scale bar 500 µm), showed thinning of LV wall and altered cardiomyocyte structure within the infarct (bottom row) following MI in ANX-A1-/- mice. (c) Increased heart weight (HW) relative to body weight (BW), (d) LV expression of the hypertrophic gene, ANP and (e) lung weight relative to body weight. Data is presented as mean ± SEM, expressed as fold change ANX-A1$^{+/+}$- sham. *P<0.05, **P<0.01, ***P<0.001 (Two-way ANOVA followed by Tukey's post-hoc test). n= 4 shams, n= 8-11 in MI groups.

Supplementary Table S1: 5’-3’ primer sequences used for gene expression by qrt-PCR.

	**Forward primer**	**Reverse primer**
**18S**	TGTTCACCATGAGGCTGAGATC	TGG TTG CCT GGG AAA ATC C
**mArg1**	TCAGAAGCTGTTCTTGGTCTGAAC	GTTCATGGGGATCCCAGTGA
**mANX-A1**	GCTGGAGAAAGGAGAAAGGG	ACACAGAGCCACCAGGATTT
**mβ-MHC**	TCT CCT GCT GTT TCC TTA CTT GCT A	GTA CTC CTC TGC TGA GGC TTC CT
**mCCR2**	GGGAACTCCTTGGTGATGCT	AGGTAGCGGTCCATGCTGAT
**mCD11c**	GCAGGAGTGTCCAAAGCAAGA	CGTGTGCTAGGTCTCTGAAGC
**mCTGF**	TGA CCC CTG CGA CCC ACA	TAC ACC GAC CCA CCG AAG ACA CAG
**mCD68**	CCAATTCAGGGTGGAAGAAA	CTCGGGCTCTGATGTAGGTC
**mFibronectin**	AAGACCATACCTGCCGAATG	CAACTGGTTGGCATGAAATG
**mFPR1**	CCTTGGCTTTCTTCAACAGC	GCCCGTTCTTTACATTGC AT
**mFPR2**	ACA GCA GTT GTG GCT TCCTT	CCT GGC CCA TGA AAA CAT AG
**mIL-1β**	TTGACGGACCCCAAAAGAT	GAAGCTGGATGCTCTCATCCTG
**mIL-10**	ACCTGGTAGAAGTGATGCCC	GGAGAAATCGATGACAGCGCC
**mMCP1**	AGGTCCCTGTCATGCTTCTG	TCTGGACCCATTCCTTCTTG
**mNLRP3**	AGGACCAGCCAGAGTGGAAT	AATGGAGATGCGGGAGAGAT
**mPeriostin**	AACCAAGGACCTGAAACACG	GTGTCAGGACACGGTCAATG
**mS100A9**	TCATCGACACCTTCCATCAA	GTCCAGGTCCTCCATGATGT
**mSRA**	CATGAACGAGAGGATGCTGACT	GGAAGGGATGCTGTCATTGAA
**mTNFα**	CTG TAG CCC ACG TCG TAG C	TTG AGA TCC ATG CCG TTG

Supplementary Table S2. Effect of LAD occlusion (1h) followed by 24h or 48h reperfusion on body weight, heart weight and systemic inflammation in WT and *ANX-A1*$^{-/-}$ mice.

	24h after myocardial I-R				48h after myocardial I-R			
	Sham		I-R		Sham		I-R	
	WT (n=4)	*ANX-A1*$^{-/-}$ (n=3)	WT (n=9)	*ANX-A1*$^{-/-}$ (n=9)	WT (n=4)	*ANX-A1*$^{-/-}$ (n=3)	WT (n=12)	*ANX-A1*$^{-/-}$ (n=10)
**Body Weight (g)**								
Pre-surgery BW(g)	32.2±1.0	27.3±1.9	31.2±1.3	29.6±0.7	30.6±0.7	26.6±2.1	30.2±0.6	26.7±0.5$^{\#\#}$
Post-surgery BW(g)	29.6±1.3	26.1±1.9	28.5±1.2	27.7±0.7	29.3±0.5	25.5±2.1	28.2±0.6	25.0±0.7$^{\#}$
Change in BW(g)	3.0±0.3	1.2±0.0	2.7±0.5	1.9±0.2	1.4±0.2	1.3±0.6	2.0±0.5	2.4±0.4
**Organ: BW (mg/g)**								
whole heart: BW	ND	ND	ND	ND	4.6±0.2	4.6±0.2	5.2±0.2	5.8±0.2
LV: BW	ND	ND	ND	ND	3.4±0.1	3.3±0.3	3.8±0.1	4.4±0.2$^{\#\#}$
RV: BW	ND	ND	ND	ND	0.9±0.1	0.8±0.1	0.9±0.1	0.9±0.1
Atria: BW	ND	ND	ND	ND	0.3±0.1	0.5±0.1	0.4±0.1	0.4±0.0
**Circulating cells/µL**								
WBC	3600±1191	2560±523	2667±470	2560±278	3613±1062	5587±180	5695±787	5108±569
Neutrophils	1715±664	997±67	1328±299	1440±294	1572±500	2153±255	2321±393	2087±379
Lymphocytes	1827±499	1513±500	1179±165	736±120	2920±738	3072±394	2634±307	2634±307
Monocytes	115±18	43±12	88±18	86±10	108±42	200±81	148±22	150±24
Basophils	0	0	9±7	40±2	2±2	27±15	43±28	34±11
Eosinophils	2±2	7±3	24±16	10±3	7±3	113±52	112±68	95±32

Data are presented as mean±SEM, numbers in brackets indicates group size. $^{\#\#}$p<0.01 vs WT I-R (Two-way ANOVA followed by Tukey's post-hoc test). BW, body weight, I-R, ischaemia-reperfusion, LV, left ventricle, RV, right ventricle, WBC, white blood cells, ND, not determined.

Supplementary Table S3. Effect of 8 days permanent occlusion on body, heart weights and systemic inflammation in WT and *ANX-A*$^{-/-}$ mice.

Data are presented as mean± SEM. *p<0.05 vs WT Permanent Occlusion (Two-way ANOVA followed by Tukey's post-hoc test). BW, body weight; ND, not detectable; RV, right ventricle; WBC, white blood cell.

	**8 Days after MI or sham**				**4 weeks after MI or sham**			
	**Sham**		**MI**		**Sham**		**MI**	
	WT	*ANX-A1*$^{-/-}$	WT	*ANX-A1*$^{-/-}$	WT	*ANX-A1*$^{-/-}$	WT	*ANX-A1*$^{-/-}$
**Body Weight (g)**								
Pre-surgery BW(g)	29.4±1.1	28.3±0.5	31.8±1.0	28.2±0.7*	30.6±2.2	31.0±0.6	29.5±0.5	28.6±0.5
Post-surgery BW(g)	28.3±1.6	27.7±0.5	30.9±0.7	27.0±1.0*	31.5±1.5	30.8±1.0	30.5±0.6	28.8±0.4
Change in BW(g)	1.1±0.7	0.6±0.2	0.7±0.4	1.2±0.4	0.9±0.8	0.3±0.9	1.0±0.3	0.1±0.3
**Organ: BW (mg/g)**								
RV:BW	0.8±0.1	0.8±0.1	0.8±0.0	1.0±0.1	0.8±0.2	0.7±0.1	0.9±0.2	0.8±0.1
Spleen: BW	3.5±0.3	3.8±0.2	3.9±0.3	4.7±0.3	3.4±0.2	3.4±0.2	3.5±0.1	3.5±0.2
Kidney: BW	6.1±0.2	6.5±0.3	6.2±0.1	6.2±0.8	6.2±0.5	6.3±0.5	6.5±0.2	6.3±0.2
**Circulating cells/μL**								
WBC	4830±75	2990±767	3991±368	4135±914	5250±465	4435±565	5739±701	5811±253
Lymphocytes	3700±580	3040±550	2834±216	2728±557	4413±416	3365±442	4605±618	4792±247
Basophils	5±5	10±7	2±1	19±18	ND	ND	ND	ND
Eosinophils	20±14	23±13	11±3	55±48	23±19	48±23	11±4	10±4

Supplementary Figure 1

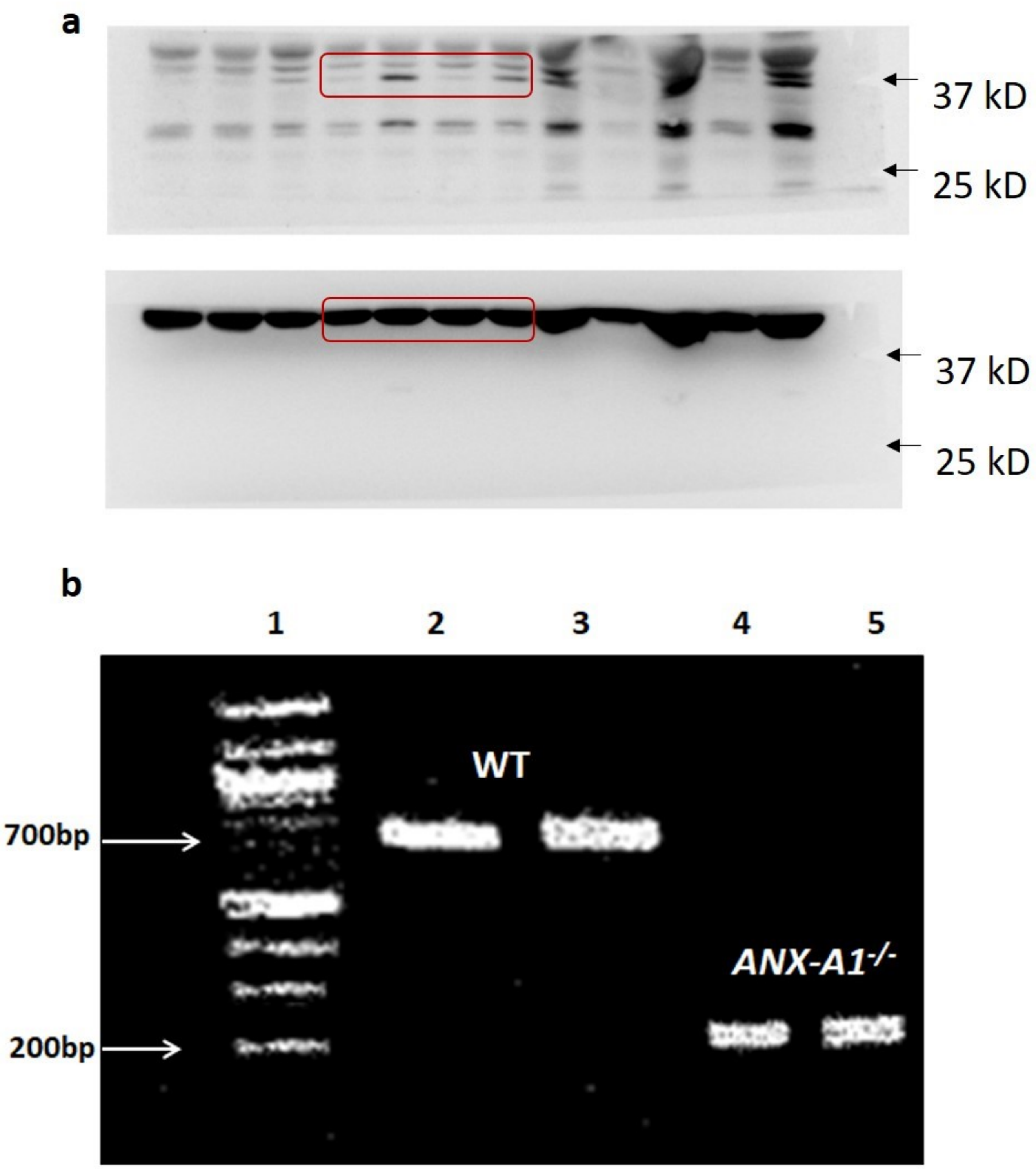

Supplementary Figure 1. Original western blots and DNA gels. (a) Expression of endogenous Anx-A1 (top) and β-actin (bottom). Molecular size markers were labeled as arrow indicated. (b) Genotyping of tail clip DNA by PCR. Lane 1: molecule weight markers; lane 2 and 3, WT mice tail DNA; Lane 4 and 5: ANX-A1-/- mice tail DNA. Note 700bp and 220bp band sizes for predicted band for the ANX-A1 and LacZ genes .DNA bands were acquired under 345nm UV light.

Supplementary Figure 2

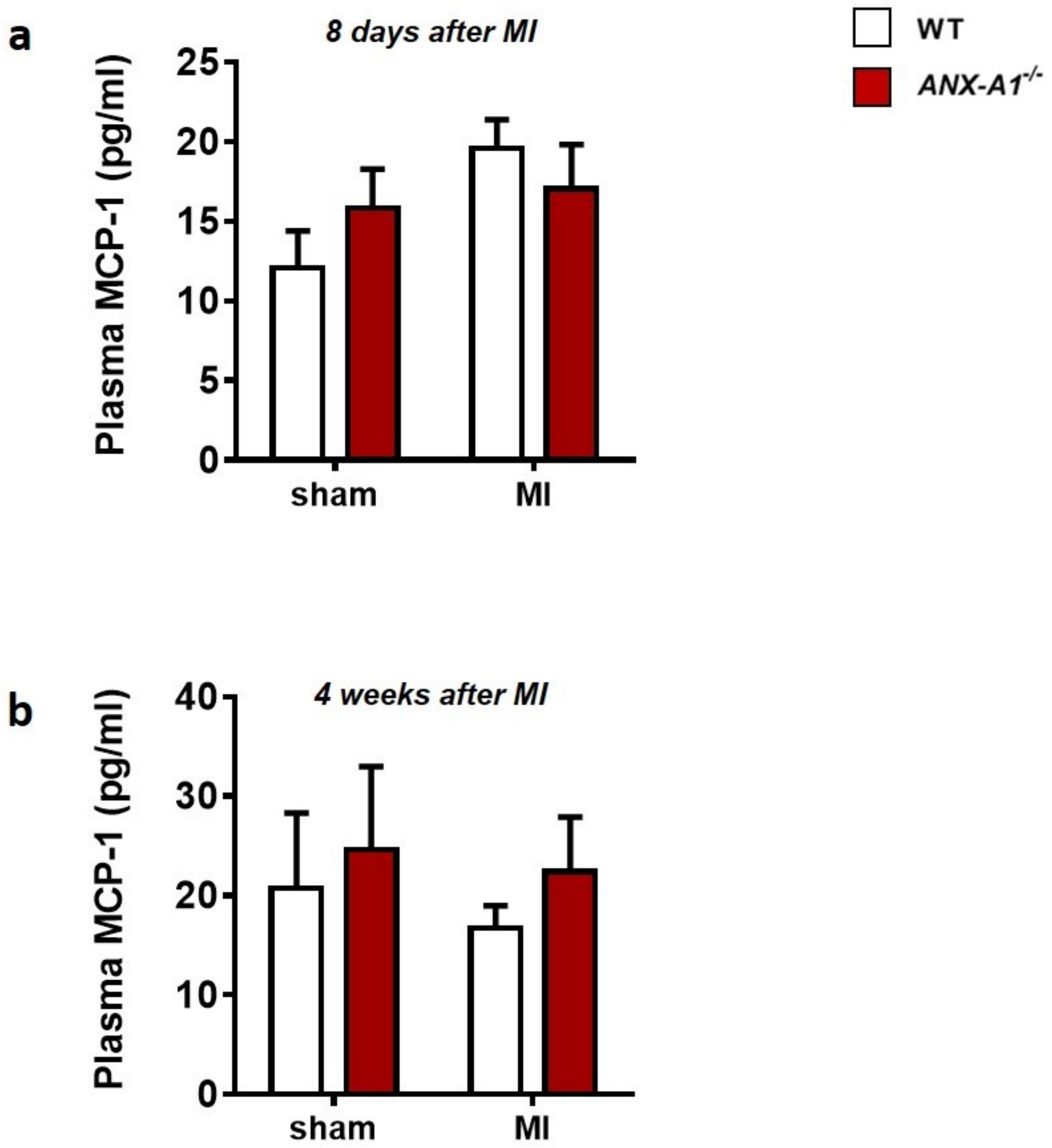

Supplementary Figure 2. Impact of deficiency of ANX-A1 on circulating MCP-1 levels after MI. (a) Plasma MCP-1 in mice from Cohort 3, after 8 days MI. (b) Plasma MCP-1 in mice from Cohort 4, after 4 weeks MI. Data is presented as mean ± SEM. P=NS (Two-way ANOVA followed by Tukey's post-hoc test). n= 3 shams, n= 6 in MI groups.

Supplementary Figure 3

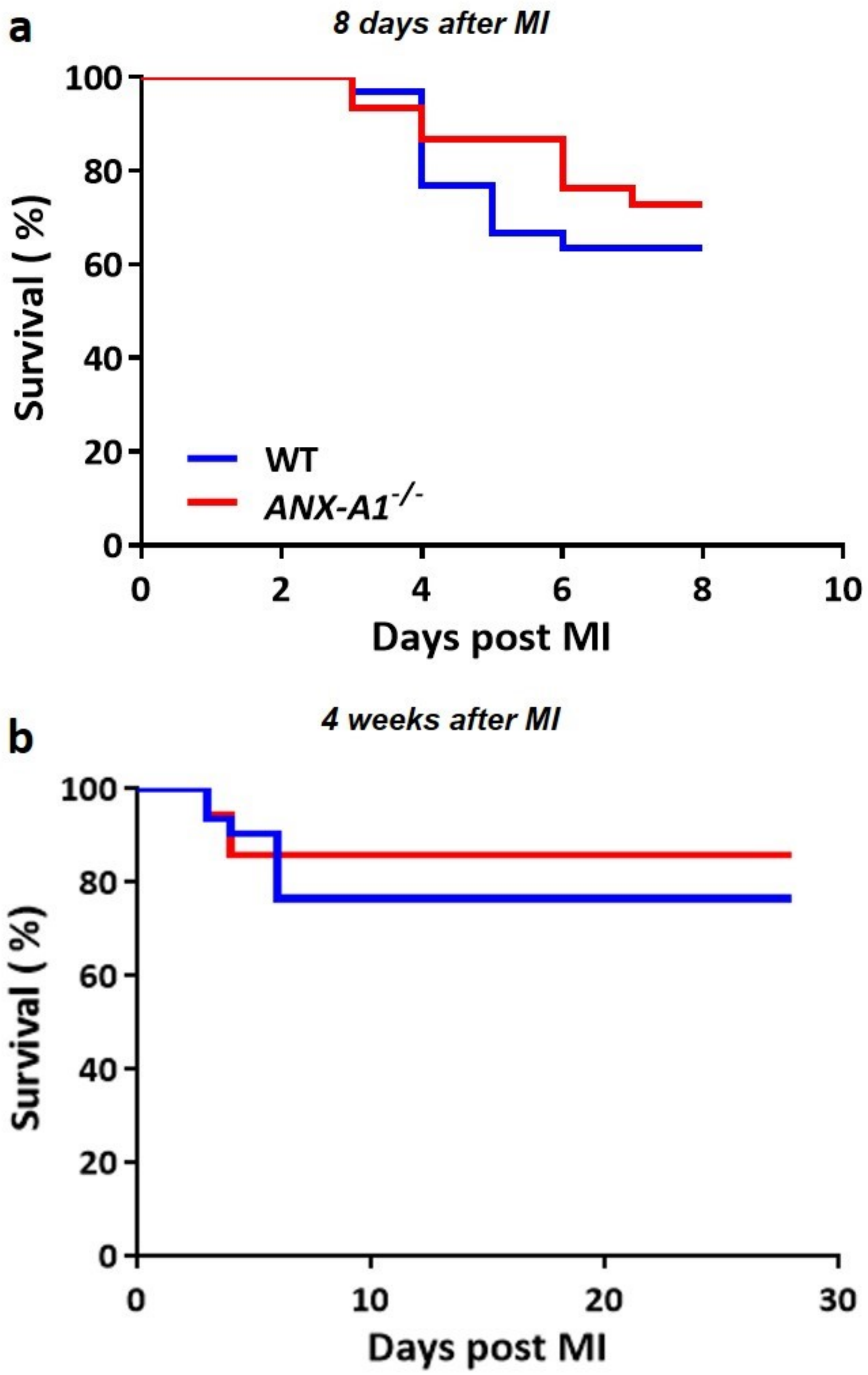

Supplementary Figure 3. Impact of deficiency of ANX-A1 on survival after MI. Day 0 represents surgery day. Peak cardiac rupture tended to occur between days 4-5 post MI. (a) Cohort 3, after 8 days MI. (b) Cohort 4, after 4 weeks MI. Log rank Mantel-COX comparison test (p=NS between genotypes). MI, myocardial infarction.

Supplementary Figure 4

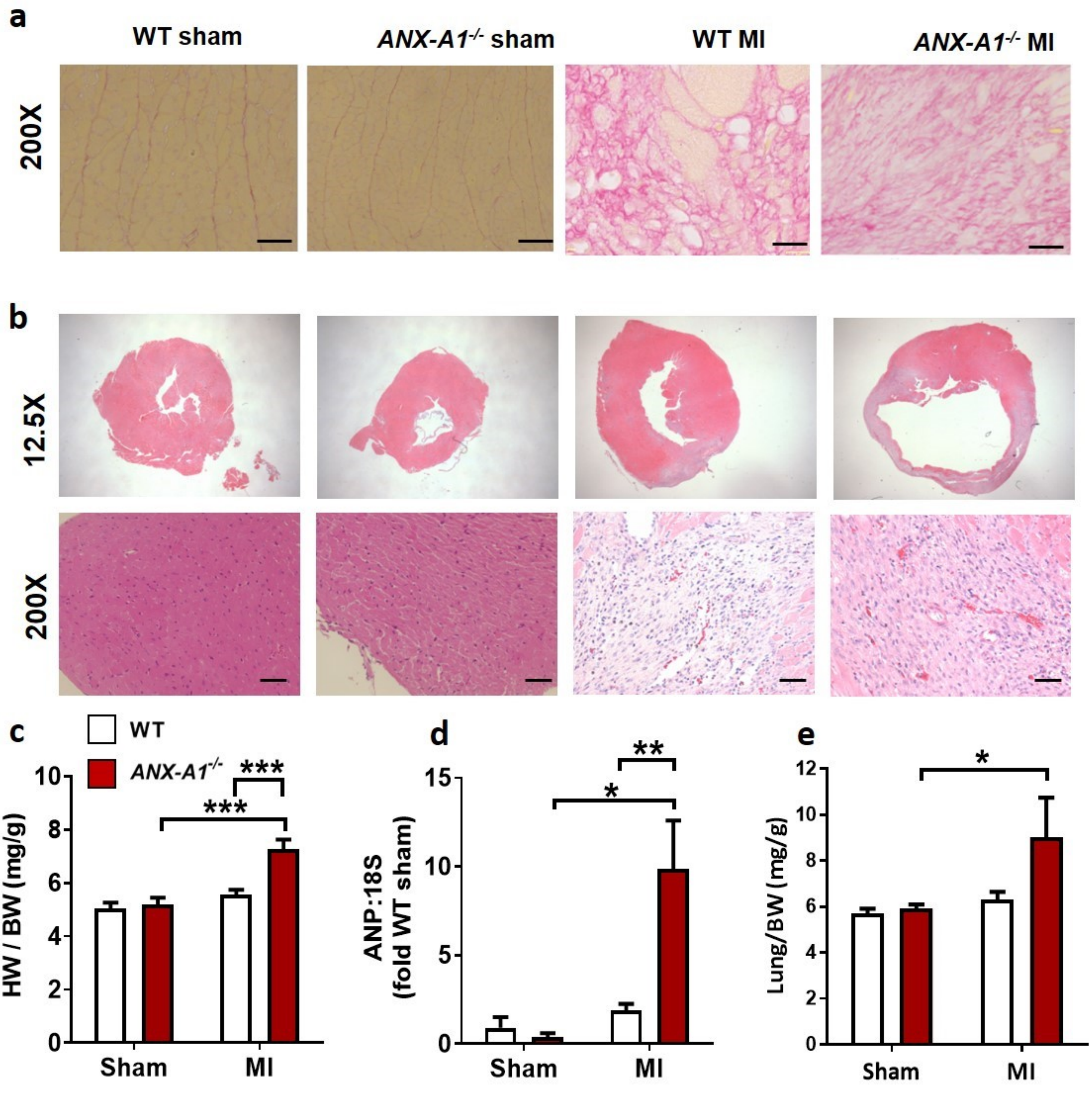

Supplementary Figure 4. Deficiency of ANX-A1 exacerbates cardiac remodelling 8 days after MI. (a) Representative images of Sirius red-stained LV sections at 200X, showing density of collagen deposition within the infarct (pink). (b) Representative images of H&E-stained LV sections (top row, scale bar 500 μm), showed thinning of LV wall and altered cardiomyocyte structure within the infarct (bottom row) following MI in *ANX-A1*$^{-/-}$ mice. (c) Increased heart weight (HW) relative to body weight (BW), (d) LV expression of the hypertrophic gene, ANP and (e) lung weight relative to body weight. Data is presented as mean ± SEM, expressed as fold change *ANX-A1*$^{+/+-}$ sham. *P<0.05, **P<0.01, ***P<0.001 (Two-way ANOVA followed by Tukey's post-hoc test). n= 4 shams, n= 8-11 in MI groups.
